# Supplementary material for: A Gene Regulatory Network Balances Neural and Mesoderm Specification during Vertebrate Trunk Development
Source: Dev Cell. 2017 May 8;41(3):243–261.e7. doi: 10.1016/j.devcel.2017.04.002 (PMC5425255; doi:10.1016/j.devcel.2017.04.002)
Supplement: Document S1. Figures S1–S4 and Table S5 [file mmc1.pdf]

Developmental Cell, Volume *41*

Supplemental Information

A Gene Regulatory Network

Balances Neural and Mesoderm Specification

during Vertebrate Trunk Development

Mina Gouti, Julien Delile, Despina Stamataki, Filip J. Wymeersch, Yali Huang, Jens Kleinjung, Valerie Wilson, and James Briscoe

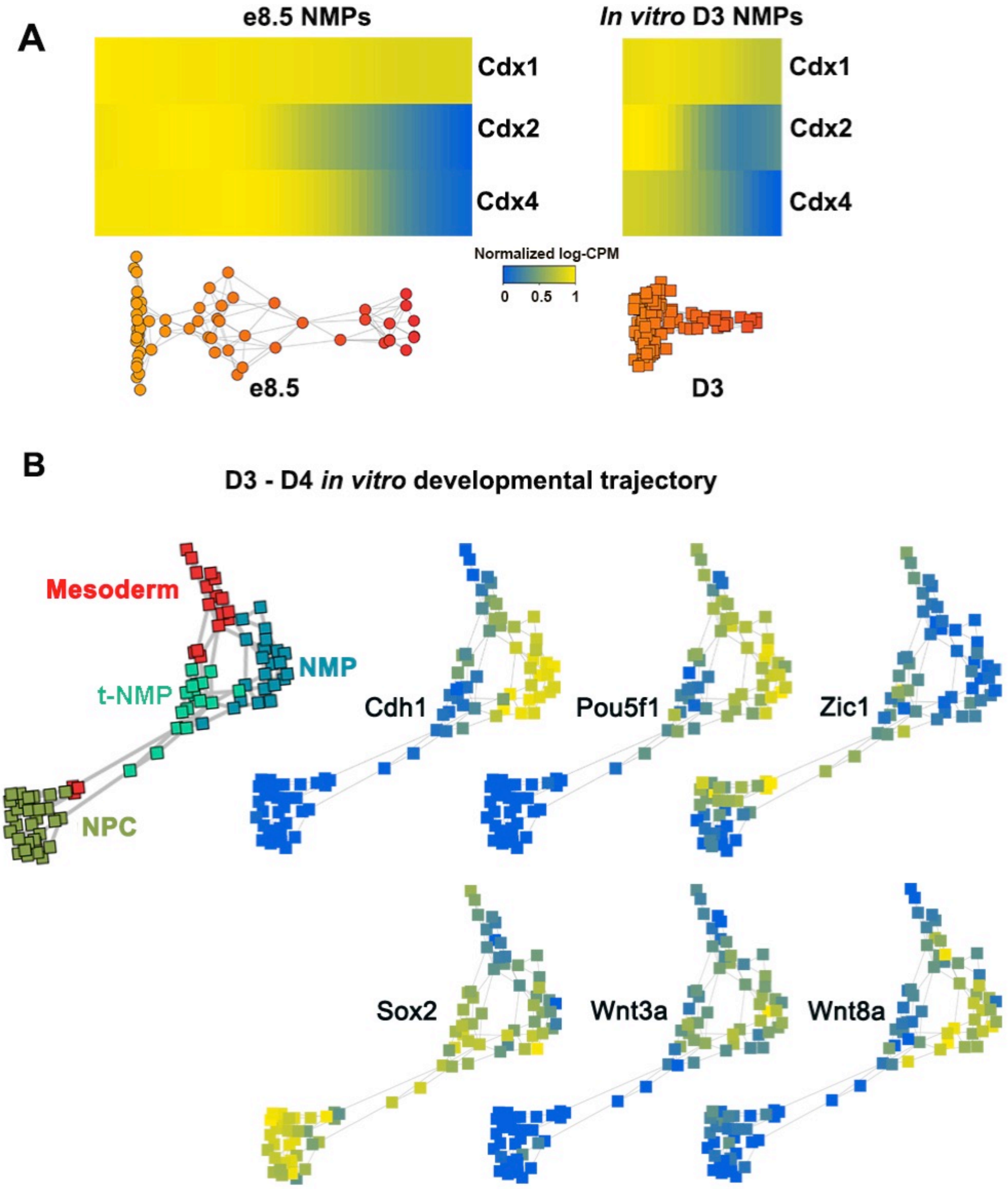

Figure S1. Temporal expression of differentially regulated genes during NMP differentiation. Related to Figure 3. (A) Comparison of *Cdx* expression (*Cdx1*, *Cdx2* & *Cdx4*) in e8.5-NMPs and *in vitro* D3 NMPs shows a similar expression pattern. *Cdx1* expression is maintained in cells differentiating to PSM both *in vivo* and *in vitro* whereas the expression of *Cdx2* and *Cdx4* is downregulated. Expression values for each gene are shown as the log of normalized counts per million reads (log-CPM). (B) *Cdh1* is specifically expressed in early NMPs (similar to e8.5-NMPs) whereas *Wnt3a* is expressed in early NMPs and t-NMPs. *Pou5f1* (*Oct3/4*) and *Wnt8a* is expressed in NMPs and is downregulated as cells progress to the t-NMP or NPC. *Zic1* and *Sox2* is upregulated as cells differentiate to NPCs.

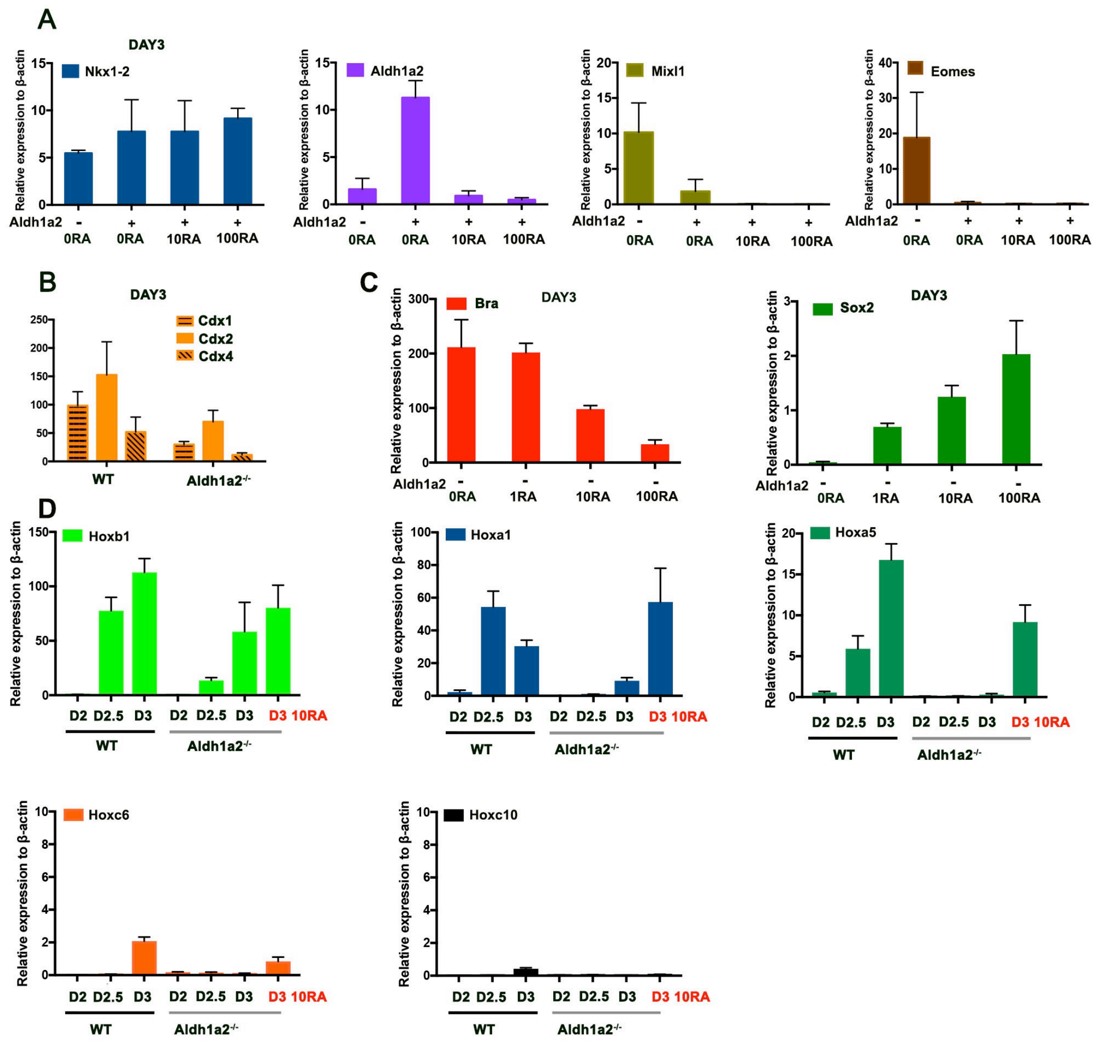

Figure S2. Induction of NMPs requires basal levels of RA signalling. Related to Figure 4. (A) qRT-PCR analysis of the expression of *Nkx1.2*, *Aldh1a2*, *Mixl1* and *Eomes* at D3 in *Aldh1a2*$^{-/-}$ and WT cells treated with CHIR/FGF or WT cells treated with CHIR/FGF/RA (RA 10nM or 100nM). Expression of *Nkx1.2* is similar in all conditions, whereas expression of *Aldh1a2* is high in WT cells at D3 but *Mixl1* and *Eomes* are induced in *Aldh1a2*$^{-/-}$ cells under CHIR/FGF conditions. (B) The expression of *Cdx1*, *Cdx2* and *Cdx4* is downregulated in the *Aldh1a2*$^{-/-}$ cells at D3. (C) qRT-PCR analysis of the expression of T/*Bra* and *Sox2* in the *Aldh1a2*$^{-/-}$ cells treated with CHIR/bFGF and different concentrations of RA (1nM, 10nM or 100nM). (D) Analysis of Hox gene expression at D2, D2.5 and D3 of differentiation revealed that anterior *Hox* genes like *Hoxb1* are induced in the absence of RA signalling (*Aldh1a2*$^{-/-}$ cells), whereas the expression of more posterior *Hox* genes like *Hoxa5* and *Hoxc6* is RA dependent. Addition of 10nM RA between D2-D3 in the *Aldh1a2*$^{-/-}$ cells rescues the expression of *Hoxa5* and *Hoxc6*.

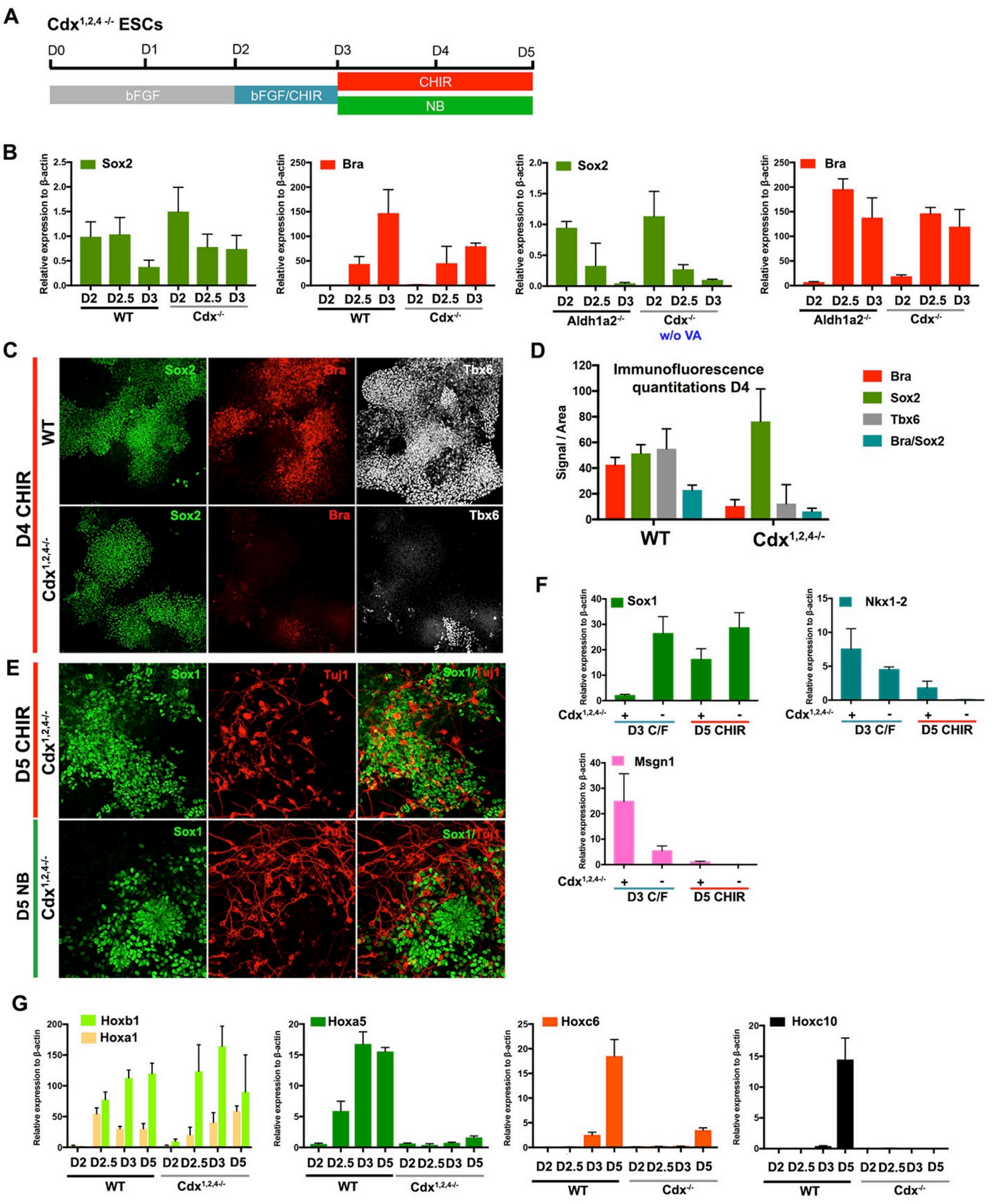

Figure S3. **Cdx genes are required for the establishment of NMP identity and induction of trunk Hox genes. Related to Figure 4.** (A) Schematic of *in vitro* differentiation conditions of *Cdx*$^{1,2,4-/-}$ cells. (B) qRT-PCR analysis of *Sox2* and *T/Bra* levels at D2, D2.5 and D3 of differentiation in WT and *Cdx*$^{1,2,4-/-}$ cells shows similar response during the early stages of differentiation (D2, D2.5) whereas at D3 the expression of *T/Bra* is significantly lower in the *Cdx*$^{1,2,4-/-}$ cells. In the absence of RA signalling (Vitamin A depleted conditions) the expression of *Sox2* is significantly reduced and *T/Bra* expression is induced at an earlier time point (D2.5) in both *Aldh1a2*$^{-/-}$ and *Cdx*$^{1,2,4-/-}$ cells. (C) At D4 of differentiation (CHIR conditions), the expression of *T/Bra* is downregulated in *Cdx*$^{1,2,4-/-}$ cells, which acquire a NPC identity. (D) Quantitation of T/Bra^{+}, Sox2^{+}, Tbx6^{+} or T/Bra^{+}/Sox2^{+} signal^{+} area normalized to DAPI area at day4 (CHIR) of differentiation in WT and *Cdx*$^{1,2,4-/-}$ cells. Error bars indicate s.d, of randomly selected independent fields n≥4. (E) At D5 *Cdx*$^{1,2,4-/-}$ cells predominantly differentiate to neural cells expressing Sox1 and Tuj1 in both CHIR and NB conditions. (F) qRT-PCR analysis of *Sox1*, *Nkx1.2* and *Msgn1* at D3 (NMP conditions) and D5 (CHIR conditions) verifies the inductions of neural identity in the *Cdx*$^{1,2,4-/-}$ cells. (G) Analysis of *Hox* gene expression at D3 and D5 (CHIR conditions) of differentiation revealed that *Cdx*$^{1,2,4-/-}$ cells express *3' Hox* genes, such as *Hoxb1*, whereas WT cells acquire a progressively more posterior identity characterized by the expression of *Hoxa5*, *Hoxc6*, *Hoxc9* and *Hoxc10* at D5.

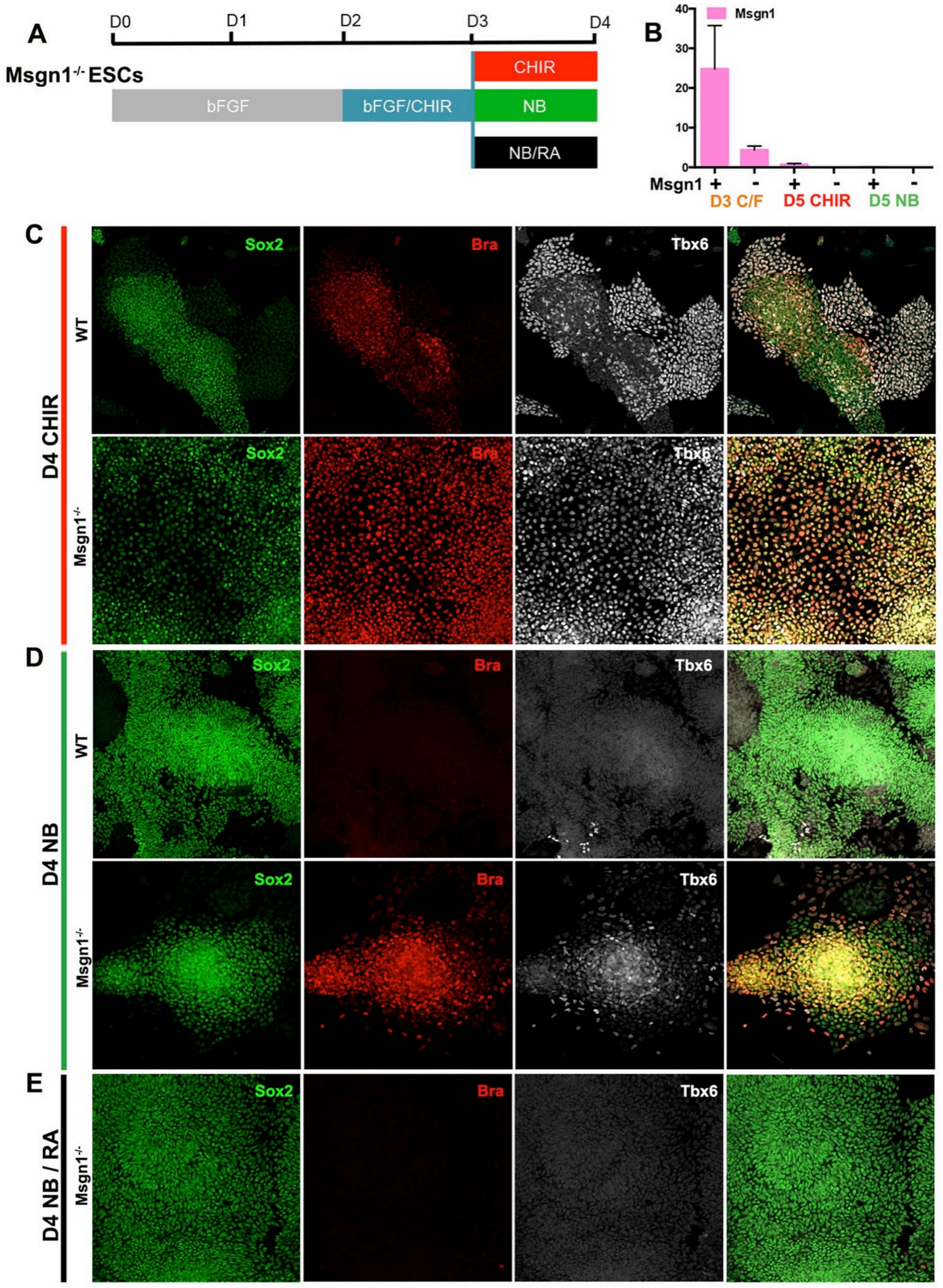

Figure S4. Msgn1 is required for the MPC to PSM transition. Related to Figure 5. (A) Schematic of *in vitro* differentiation conditions of *Msgn1*$^{-/-}$ cells. (B) Expression of *Msgn1* is induced at D3 in the WT cells exposed to CHIR/FGF. (C) At D4 in CHIR conditions, *Msgn1*$^{-/-}$ cells co-express T/Bra/Sox2 with Tbx6 whereas WT cells express Tbx6 but not T/Bra. (D) After removal of CHIR/FGF, in NB conditions, the expression of T/Bra is maintained longer in the *Msgn1*$^{-/-}$ cells compared to WT. Treatment with 100nM RA from D3 to D4 resulted in the downregulation of T/Bra in *Msgn1*$^{-/-}$ cells.

Table S5. Oligonucleotides used in the study, related to STAR Methods.

**Gene**	**Primer Sequence (5’-3’)**
Actin F	TGGCTCCTAGCACCATGA
Actin R	CCACCGATCCACACAGAG
Eomes F	GGCCTACCAAAACACGGATATC
Eomes R	TTTCTGAAGCCGTGTACATGGA
Mixl1 F	CTACCCGAGTCCAGGATCCA
Mixl1 R	ACTCCCCGCCTTGAGGATAA
Nkx1.2 F	ACTGCCTTCACTTACGAGCA
Nkx1.2 R	AAATTTTGACCTGCGTCTCC
T/Bra F	ACACACGGCTGTGAGAGGTA
T/Bra R	TTATCATGGGACTGCAGCAT
Cdx1 F	ACAGCCGGTACATCACTATCC
Cdx1 R	CTTGTTTACTTTGCGCTCCTTG
Tbx6 F	CCCAACTATGCAGCCAACACT
Tbx6 R	CTGTGTGATCCTAGGGTTCTGGTA
Msgn1 F	CTTCTGACACCGCTGGTCTG
Msgn1 R	GTGACTGCCGTAGCCATCG
Hoxb1 F	AGAGGCTGGCTTACGAGAC
Hoxb1 R	GGTTGAGGCTTGCTTGAGG
Hoxc6 F	CAGGTAAAGGCAAAGGGATG
Hoxc6 R	ATAGGCGGTGGAATTGAGG
Hoxc9 F	AGCCGACAGAGACAGATTAC
Hoxc9 R	AATGCCAGTCCCAGAAGC
Hoxc10 F	GTCCAGACACCTCGGATAAC
Hoxc10 R	AATGGTCTTGCTAATCTCCAG
Hoxa13 F	AAATGTACTGCCCCAAAGAG
Hoxa13 R	GATATCCTCCTCCGTTTGTC
Hoxc13 F	GTCAGGTGTACTGCTCCAAG
Hoxc13 R	CCTTCTCTAGCTCCTTCAGC
Cdx2 F	TAGTCGATACATCACCATCAGG
Cdx2 R	TGATTTTCCTCTCCTTGGCTCT
Cdx4 F	GCAATAGATACATCACCATCAGG
Cdx4 R	ACTTTGCACGGAACCTCCAG
Fgf8 F	GCTCATTGTGGAGACCGATA
Fgf8 R	AATACGCAGTCCTTGCCTTT
Wnt3a F	ATGGCTCCTCTCGGATACCT
Wnt3a R	GGGCATGATCTCCACGTAGT
Aldh1a2 F	ATGGGTGAGTTTGGCTTACG
Aldh1a2 R	GGTTCATTGGAAGGCAGAAA
Sox1 F	AGCGTGCCTTTGATTTCTCT
Sox1 R	GGGATAAGACCTGGGTGAGA
Cyp26a1 F	GCAGGAAATACGGCTTCATC
Cyp26a1 R	ATCACCTTCTTTCGCTGCTT
Sox2 F	GGCAGCTACAGCATGATGCAGGAGC
Sox2 R	CTGGTCATGGAGTTGTACTGCAGG
