# Supplementary material for: A Gene Regulatory Network Balances Neural and Mesoderm Specification during Vertebrate Trunk Development
Source: Dev Cell. 2017 May 8;41(3):243–261.e7. doi: 10.1016/j.devcel.2017.04.002 (PMC5425255; doi:10.1016/j.devcel.2017.04.002)
Supplement: Document S2. Article plus Supplemental Information [file mmc6.pdf]

Developmental Cell

Article

A Gene Regulatory Network Balances Neural and Mesoderm Specification during Vertebrate Trunk Development

Graphical Abstract

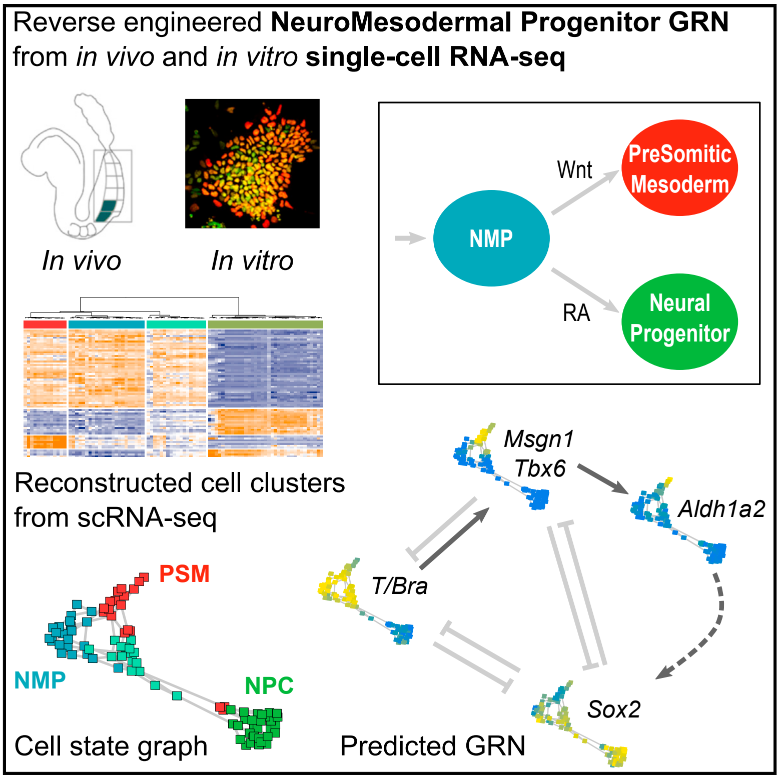

Highlights

- Single-cell RNA-seq reveals a signature of neuromesodermal progenitors
- In vitro NMPs resemble and differentiate similar to their in vivo counterparts
- Dual role for retinoic acid signaling in NMP induction and neural differentiation
- A transcriptional network regulates neural versus mesodermal allocation

Authors

Mina Gouti, Julien Delile, Despina Stamataki, ..., Jens Kleinjung, Valerie Wilson, James Briscoe

Correspondence

mina.gouti@mdc-berlin.de (M.G.), james.briscoe@crick.ac.uk (J.B.)

In Brief

Neuromesodermal progenitors (NMPs) generate cells of the spinal cord and somites. Gouti et al. demonstrate that in vitro NMPs resemble in vivo counterparts at the single-cell level and define a regulatory network that balances the generation of neural and mesodermal tissue to facilitate orderly extension of the embryonic axis.

Gouti et al., 2017, Developmental Cell *41*, 243–261
May 8, 2017 © 2017 The Author(s). Published by Elsevier Inc.
http://dx.doi.org/10.1016/j.devcel.2017.04.002

Developmental Cell
Article

A Gene Regulatory Network Balances Neural and Mesoderm Specification during Vertebrate Trunk Development

Mina Gouti,[1,3,4,*] Julien Delile,[1,4] Despina Stamataki,[1] Filip J. Wymeersch,[2] Yali Huang,[2] Jens Kleinjung,[1] Valerie Wilson,[2] and James Briscoe[1,5,*]
[1]The Francis Crick Institute, 1 Midland Road, London, NW1 1AT, UK
[2]MRC Centre for Regenerative Medicine, Institute for Stem Cell Research, School of Biological Sciences, University of Edinburgh, 5 Little France Drive, Edinburgh EH16 4UU, UK
[3]Max Delbrück Center for Molecular Medicine, Robert-Rössle-Strasse 10, 13125 Berlin, Germany
[4]These authors contributed equally
[5]Lead Contact
*Correspondence: mina.gouti@mdc-berlin.de (M.G.), james.briscoe@crick.ac.uk (J.B.)
http://dx.doi.org/10.1016/j.devcel.2017.04.002

SUMMARY

Transcriptional networks, regulated by extracellular signals, control cell fate decisions and determine the size and composition of developing tissues. One example is the network controlling bipotent neuromesodermal progenitors (NMPs) that fuel embryo elongation by generating spinal cord and trunk mesoderm tissue. Here, we use single-cell transcriptomics to identify the molecular signature of NMPs and reverse engineer the mechanism that regulates their differentiation. Together with genetic perturbations, this reveals a transcriptional network that integrates opposing retinoic acid (RA) and Wnt signals to determine the rate at which cells enter and exit the NMP state. RA, produced by newly generated mesodermal cells, provides feedback that initiates NMP generation and induces neural differentiation, thereby coordinating the production of neural and mesodermal tissue. Together, the data define a regulatory network architecture that balances the generation of different cell types from bipotential progenitors in order to facilitate orderly axis elongation.

INTRODUCTION

Cell fate decisions in developing tissues are made by gene regulatory networks comprising transcription factors and intercellular signals. These networks determine the rate of self-renewal and differentiation to ensure the balanced generation of different cell types and the production of well-proportioned tissues (Stern et al., 2006; Davidson, 2010). The formation of the vertebrate trunk, which extends progressively during embryogenesis, is one example. Successively more posterior neural and paraxial presomitic mesodermal (PSM) cells of the trunk are generated from a bipotential population of cells (Tzouanacou et al., 2009), termed neuromesodermal progenitors (NMPs) at the posterior end of the embryo. Proliferation of NMPs fuels the elongation of axial tissues (Cambray and Wilson, 2002; 2007; Wilson et al., 2009; Henrique et al., 2015; Neijts et al., 2014; Kimelman, 2016). Hence, the rate at which NMPs are generated, self-renew, and differentiate must be carefully regulated in order to balance the production of different trunk tissues and to prevent the premature or delayed depletion of NMPs that will affect the length of the embryo.

NMPs reside in the node-streak border (NSB), caudal lateral epiblast (CLE) and the chordoneural hinge (CNH) of elongating embryos (Cambray and Wilson, 2002; 2007; Delfino-Machin et al., 2005; Kondoh and Takemoto, 2012; Olivera-Martinez et al., 2012). These regions express Wnt and FGF ligands (Wilson et al., 2009). Interfering with either signal results in the depletion of NMPs and the premature truncation of the body axis (Takada et al., 1994; Wilson et al., 2009; Yoshikawa et al., 1997; van de Ven et al., 2011). Both Wnt and FGF signaling are implicated in posteriorizing cells by inducing the *Cdx* transcription factors (TFs) that promote the expression of more posterior *Hox* genes (van den Akker et al., 2002; Nordstrom et al., 2006; van de Ven et al., 2011; Mazzoni et al., 2013; Neijts et al., 2016; Amin et al., 2016). Moreover, Wnt signaling is required for the differentiation of NMPs to mesodermal tissue (Martin and Kimelman, 2012; Garriock et al., 2015) and the loss of Wnt3a results in a depletion of mesodermal tissue in both mice and zebrafish (Yoshikawa et al., 1997; Martin and Kimelman, 2008; Garriock et al., 2015).

Primitive streak and node cells transiently express the retinoic acid (RA)-synthesizing enzyme *Aldh1a2* (Ribes et al., 2009). Mouse embryos lacking *Aldh1a2* are truncated, suggesting a role for RA in axis elongation (Niederreither et al., 1999; Cunningham et al., 2015; Niederreither and Dolle, 2008; Duester, 2008) nevertheless, the role of RA in the establishment of NMPs remains unclear. At later stages of development, RA emanating from *Aldh1a2*-expressing somitic cells promotes the expression of genes characteristic of neural progenitors in the spinal cord. In addition, RA inhibits expression of both *Wnt3a* and *Fgf8*, and exposure of the tail region to increased concentrations of RA can arrest axis elongation (Olivera-Martinez et al., 2012). Excess RA concentration in the tail bud is prevented by the RA-metabolizing enzyme *Cyp26a1*, which is induced by *Cdx* genes and

This is an open access article under the CC BY license (http://creativecommons.org/licenses/by/4.0/).

T/Brachyury under the control of Wnt/Fgf signaling (Martin and Kimelman, 2010; Vidigal et al., 2010; Savory et al., 2009; Young et al., 2009; van Rooijen et al., 2012). However, the overlapping functions and proximity of events hindered assigning direct and indirect activities to individual signaling pathways (Kimelman, 2016; Henrique et al., 2015; Gouti et al., 2015; Neijts et al., 2014).

The co-expression of the TFs T/Brachyury (T/Bra) and Sox2 is characteristic of NMPs (Olivera-Martinez et al., 2012; Gouti et al., 2014; Tsakiridis et al., 2015; Wymeersch et al., 2016). Both Wnt and FGF signaling have been implicated as inducers of *T/Bra* in NMPs (Yamaguchi et al., 1999; Martin and Kimelman, 2008). Moreover, Cdx-binding regions have been found upstream of the T/Bra gene (Savory et al., 2009). This and subsequent analysis (van Rooijen et al., 2012) has led to the suggestion that Cdx proteins, induced by Wnt signaling, maintain T/Bra expression in NMPs, but are dispensable for its initial induction. However, Cdx proteins also appear to regulate Wnt and FGF expression in NMPs (Young et al., 2009; Savory et al., 2009; van Rooijen et al., 2012), thus the loss of *T/Bra* expression in the absence of Cdx might be due to the loss of these signals and the depletion of NMPs.

Neural cells differentiating from NMPs downregulate *T/Bra* but maintain *Sox2* expression (Gouti et al., 2014, 2015; Tsakiridis and Wilson, 2015; Gouti et al., 2015). By contrast, as NMPs differentiate into mesoderm, expression of *Sox2* is downregulated and *Msgn1* and *Tbx6* are upregulated to form nascent mesodermal progenitor cells (MPCs) (Chalamalasetty et al., 2011). Then, as cells commit to a PSM identity, expression of *T/Bra* is downregulated. In embryos lacking *T/Bra*, mesoderm induction fails and axis elongation halts (Herrmann et al., 1990). This is, at least in part, explained by a requirement for *T/Bra* for the induction of *Msgn1* and *Tbx6* (Yamaguchi et al., 1999; Yabe and Takada, 2012). Moreover, the loss of PSM tissue in the absence of *Tbx6* or *Msgn1* is accompanied by ectopic generation of neural tissue (Chapman and Papaioannou, 1998; Yoon et al., 2000; Chalamalasetty et al., 2014), raising the question of the role that the induction of these TFs plays in balancing neural and mesodermal production from NMPs.

Taken together, the data suggest complex regulatory mechanisms with multiple interactions and feedbacks. It has proven challenging, however, to assemble a definitive network that explains the generation of NMPs and their balanced allocation toward mesodermal and neural tissue. These difficulties arise from the necessity of analyzing in vivo experimental perturbations in which axis elongation fails or the expression of signals is lost. To circumvent this, we have taken advantage of the in vitro directed differentiation that we and others have recently developed to generate NMPs from pluripotent stem cells (Gouti et al., 2014; Tsakiridis et al., 2015; Turner et al., 2014; Lippmann et al., 2015). This system decouples the development of NMPs and trunk cell types from the specific tissue architecture associated with axis elongation, thereby avoiding the difficulty of interpreting data from chimeric or morphologically abnormal embryos. Furthermore, it allows exogenous control of the supply and timing of signaling molecules. Thus, aspects of the gene regulatory network that are tightly linked in vivo can be separated and assayed in vitro.

Using single-cell transcriptome analysis we first established the similarity between in vivo and in vitro derived NMPs. We then reverse engineered the transcriptional network responsible for NMP induction and differentiation. This revealed a network comprising the TFs *Cdx1, 2, 4*, *T/Bra*, *Sox2*, *Msgn1*, and *Tbx6*, which integrate Wnt and RA signaling to regulate entry to and exit from the NMP state. Mutation of individual or multiple components validated the network. Within the network, RA plays dual roles. Initially, RA is required for the expression of *Sox2* and generation of NMPs; later, increased levels of RA drive neural differentiation. *Cdx* genes not only posteriorize cells but also, by restraining RA signaling, maintain *T/Bra* expression to allow mesoderm induction. *Msgn1* and *Tbx6* control the timing and outcome of NMP differentiation by cell-autonomously repressing T/*Bra* and *Sox2* to propel mesoderm differentiation and by inducing the RA-producing enzyme *Aldh1a2*. The latter increases RA levels to non-autonomously promote neural differentiation and thereby provides regulative feedback that balances neural and mesoderm production. A dynamic model capturing these interactions demonstrates how the network coordinates the generation of the two cell types from the bipotential progenitor and ensures the well-proportioned generation of tissues necessary to build the vertebrate trunk.

RESULTS

Single-Cell Transcriptome Analysis of NMP Cells from Mouse Embryos

NMPs are a transient population, arising early in gastrulation, located in the CLE and CNH at the anterior end of the receding primitive streak and tail bud (Cambray and Wilson, 2002; 2007; Wymeersch et al., 2016; Olivera-Martinez et al., 2012). They can be identified in vivo by the co-expression of the TFs *Sox2* and *T/Bra* (Tsakiridis et al., 2014; Martin and Kimelman, 2012; Olivera-Martinez et al., 2012). However, defining a definitive molecular signature has proved challenging because of their scarcity and transience in vivo. To address this, we performed single-cell transcriptome analysis (RNA sequencing [RNA-seq]) of NMPs micro-dissected from mouse embryos at two developmental stages (e8.5 and e9.5) (Figure 1A). We define these as embryo NMPs (e-NMPs).

Following sequencing and mapping of transcriptomes of dissected cells, we applied quality filters (See STAR Methods) that resulted in data from a total of 128 single e-NMP transcriptomes being retained for subsequent analysis (62 cells from e8.5 and 66 cells from e9.5). We took an unbiased, data-driven approach (STAR Methods). To establish concerted patterns of expression within an initial set of ~11,000 genes we analyzed those genes whose expression level distribution showed sufficient statistical dependence with at least two other genes. We used mutual information (MI) (reviewed in Mc Mahon et al., 2014) to measure these relationships with a cut-off value MI > 0.25. Hierarchical clustering of these 136 genes produced five major groups (modules) representing distinct patterns of gene expression. We examined the genes in each module (Table S1). Module 1 contained genes expressed in e8.5 primitive streak and e8.5 NMPs, including *Cdh1*, *Cdx1*, and *Fst*; module 2 comprised genes associated with e9.5 NMPs including *Cyp26a1* and posterior *Hox* genes; module 3 was enriched for genes related to PSM specification including

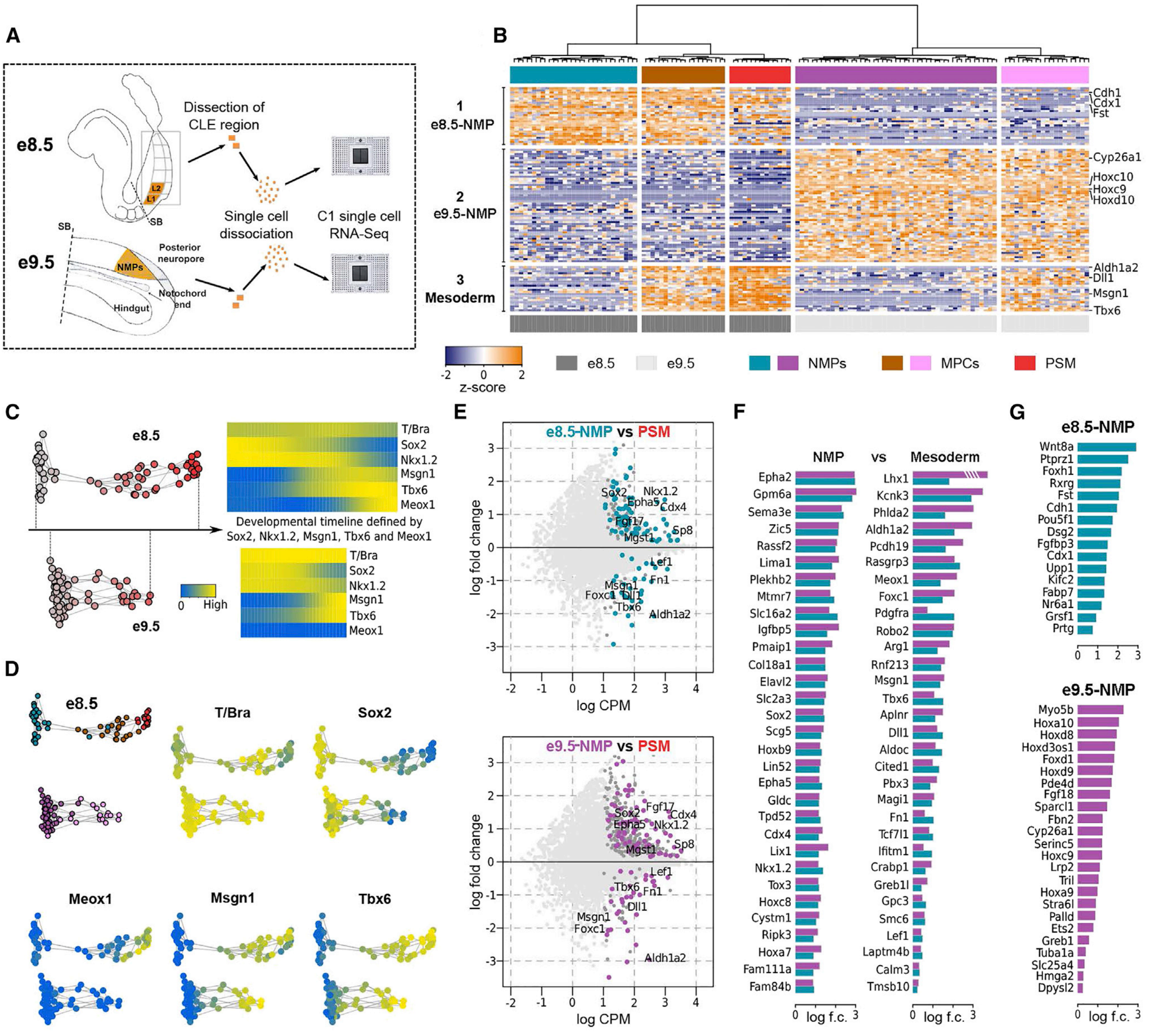

Figure 1. Single-Cell Transcriptome Analysis of In Vivo NMPs Defines the Molecular Signature of e8.5 and e9.5 NMPs

(A) Strategy for single-cell transcriptional analysis of e-NMPs dissected from the CLE region of e8.5 and e9.5 mouse embryos.

(B) Hierarchical clustering of embryo-derived single cells, using the genes in the first three modules (Table S1), columns represent cells and rows correspond to genes. This separates cells into two large groups correlating with developmental age (e8.5 and e9.5). Within the e8.5 group, three smaller clusters could be distinguished, an e8.5 NMP identity (expressing genes in module 1), MPCs (expressing genes in modules 1 and 3) and mesodermal cells (Meso) (expressing genes in module 3). The e9.5 group was subdivided into two groups, one associated with e9.5 NMP identity (module 2) the other with MPC fate (modules 2 and 3). The cluster identity of each cell from the e8.5 and e9.5 embryos is indicated in orange (e8.5 NMPs), purple (e9.5 NMPs), brown and pink (MPCs), and red (Meso).

(C) Pseudotemporal ordering of cells (right) obtained via the associated cell state graph (left) (expression levels are indicated as normalized counts per million reads). The white-to-red colors indicate NMP-to-mesodermal fractional identity of each cells defined by *Sox2*, *Nkx1.2*, *Msgn1*, *Tbx6*, and *Meox1* levels.

(D) Developmental trajectories of single cells from e8.5 and e9.5 mouse embryos reveals three distinct populations, NMP (T/Bra^{+}/Sox2^{+}), MPC (T/Bra^{+}/Msgn1^{+}/Tbx6^{+}), and PSM (T/Bra^{-}/Msgn1^{+}/Tbx6^{+}).

(E) Molecular signature of e-NMP cells identified by differential expression analysis of the e8.5 (top) and e9.5 (bottom) NMPs with PSM cells.

(F) The analysis identified 68 genes that were associated with both e8.5 and e9.5 NMP identity and 31 genes associated with mesodermal differentiation. For clarity we have shown only the 31 genes most enriched in e-NMPs. The complete gene lists are given in Table S2.

(G) Differential expression analysis of e8.5 NMPs and e9.5 NMPs defines the molecular signature of e-NMPs at different developmental stages. Log CPM, logarithmic counts per million; Log f.c., logarithmic fold change; NMPs, neuromesodermal progenitors; MPC, mesodermal progenitors; PSM, presomitic mesoderm; SB, somite border.

Msgn1, *Tbx6*, *Aldh1a2*, and *Dll1*. Finally, modules 4 and 5 included genes implicated in RNA processing, metabolic processes, ribosome generation, as well as some chromatin components.

Using the three modules that comprised genes characteristic of the primitive streak, NMPs and PSM, we performed hierarchical clustering to group the cells (Figure 1B). This divided the cells into two large groups, which correlated with embryonic age. Cells from e8.5 had high levels of activity of genes in module 1 (e8.5 NMPs), whereas module 2 genes were expressed at high levels in e9.5 NMP cells. Within these divisions, cells were further grouped resulting in a total of five clusters. The activity of genes in these clusters suggested that at both embryonic ages a subset of cells expressed PSM-associated genes contained in module 3. The presence of PSM cells in both e8.5 and e9.5 isolated e-NMPs was expected due to the close physical proximity of these populations in the mouse embryo and the dissection recovering cells from both populations. Taken together the analysis suggests that the dissection captures e-NMPs and cells differentiating toward PSM.

Given the presence of NMPs and their PSM progeny in the dataset, we asked whether it was possible to reconstruct a timeline of e-NMP differentiation. For this we used the 105 genes contained in the three modules (Table S1). Consensus graphs for e8.5 and e9.5 datasets were constructed using frequently occurring edges in minimum spanning trees from multiple randomized subsets of the data (see STAR Methods). This resulted in hypothetical developmental trajectories, pseudotemporal orderings, for the e8.5 and e9.5 cells (Figure 1C). We explored these timelines by assessing the expression of several TFs with known expression behaviors during the differentiation of NMPs to PSM. In both the e8.5 and e9.5 timelines, cells at one extremity had high levels of the NMP expressed genes, *Sox2*, *T/Bra*, *Cdx2*, and *Cdx4*. At the other end there were lower levels of *Sox2*, *Cdx2*, and *Cdx4*, but high levels of the PSM determinants *Msgn1* and *Tbx6* (Figures 1C, 1D, and S1A). This is consistent with the known changes in gene expression that accompany the differentiation of NMP to PSM, and indicates that we had successfully reconstructed the trajectory of NMP to PSM differentiation. We term these reconstructed trajectories "developmental timelines".

The developmental timelines revealed three developmentally distinct groups of cells. First, a group of cells, present at both e8.5 and e9.5 that have the characteristics of NMPs, these expressed *T/Bra*, *Sox2*, *Nkx1.2*, *Cdx2*, and *Cdx4*. Second, a transition population that expressed *T/Bra* and had upregulated *Msgn1* and *Tbx6*, in which *Sox2* and *Nkx1.2* expression was still detectable albeit at decreasing levels. This is present at both e8.5 and e9.5 and we label these MPCs after the population defined by Chalamalasetty et al., 2014. Finally, a population of cells, present at e8.5 but not in the e9.5 that expressed *Msgn1*, *Tbx6*, and *Meox1*, in which *Nkx1.2* and *Sox2* had been repressed (Figures 1B–1D). This signature is characteristic of PSM cells (Chalamalasetty et al., 2014). The absence of cells with PSM identity from the e9.5 sample reflects the anatomical differences between the two time points and the region of the embryo included in the dissections at the two time points (Figure 1A).

The data allowed us to define a molecular signature of e-NMP cells. We performed differential expression analysis of the e8.5 and e9.5 NMP cells (labeled cerulean and purple, respectively) by comparing them with more differentiated (PSM) cells (Figure 1E). This analysis identified 99 genes differentially expressed in both e8.5 and e9.5 NMP populations (Table S2). Out of these, 31 genes were expressed at lower levels in e-NMP cells, compared with mesoderm, and were mainly associated with PSM differentiation, such as *Aldh1a2*, *Meox1*, *Dll1*, and *Cited1*. The other 68 differentially expressed genes were expressed more highly in e-NMPs. These included *Nkx1.2*, *Sox2*, *Cdx2*, *Cdx4*, and *Sp8*, described previously as expressed in NMPs, but also other genes potentially functioning in NMPs such as *Epha2*, *Epha5*, *Sema6a*, or *Zic5*. This set of genes thus provides an in vivo molecular signature of e-NMPs (Figure 1F).

We next identified genes differentially regulated between the e8.5 and e9.5 NMP populations, which represents change over embryonic time. *Cadherin* (*Cdh1*), *Follistatin* (*Fst*), and *Pou5f1* (*Oct3/4*) were specifically expressed in e8.5 NMPs as was, for example, *Grsf1*, a gene implicated in axial elongation (Lickert et al., 2005) (Figure 1G). By contrast, *Cyp26a1*, an RA-degrading enzyme that functions as a negative feedback regulator of RA, was expressed at higher levels in e9.5 NMPs than in e8.5 NMPs (Figure 1G). However, the most striking difference between the e8.5 and e9.5 NMPs was the expression of *Hox* genes. The e9.5 NMP cells expressed significantly higher levels of posterior *Hox* genes (*Hox 6–10*), consistent with the acquisition of more posterior axial identity and a contribution to more posterior trunk regions as development progresses. The data allow the reconstruction of a developmental timeline of e-NMP to PSM differentiation and an embryonic aging timeline (associated with the axial identity of NMPs) for the transition from e8.5 to e9.5 NMPs.

In Vitro Derived NMPs Resemble Their In Vivo Counterparts

We have recently described the generation of NMP cells from mouse embryonic stem cells (ESCs) (Gouti et al., 2014). We generated single-cell transcriptomes of in vitro derived NMPs at day 3 (D3) of differentiation (Figure 2A) and compared these to their in vivo counterparts (e-NMPs) (Figure 1A). We took advantage of the gene signatures defined in vivo and used the three modules to characterize the in vitro NMPs (Figure 2B). A t-distributed stochastic neighbor embedding (tSNE) projection of e8.5, e9.5 and D3 NMPs, using the genes comprising the three modules, indicated that D3 NMPs were more similar to e8.5 NMPs than e9.5 NMPs (Figure 2D). Moreover, hierarchical clustering separated the in vitro NMPs into three groups (Figure 2B). Consistent with the tSNE projection, examination of the activity of each of the genes modules indicated that the majority (65%) of D3 NMPs were similar to e8.5 NMPs. In addition, approximately 10% of the cells had a gene expression profile similar to e9.5 NMPs, and the remaining ~25% appeared to be PSM (Figure 2C).

We constructed a pseudotemporal ordering of D3 NMPs employing the 99 genes comprising the in vivo e-NMP signature (Table S2). Analysis of differentially expressed genes along the NMP trajectory recovered many of the same genes that displayed differential expression along the in vivo e8.5

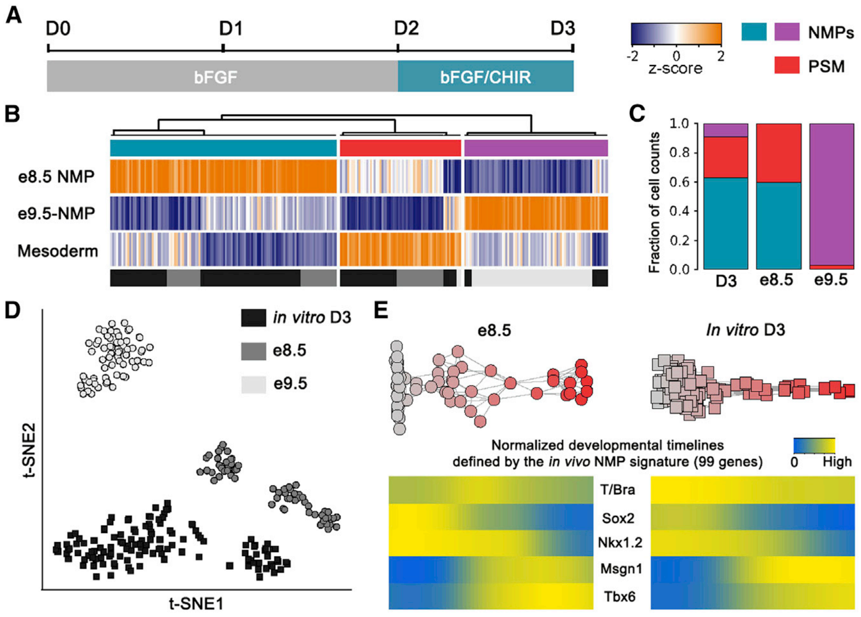

Figure 2. Single-Cell Analysis of In Vitro Derived NMPs Indicates that They Resemble Their In Vivo Counterparts

(A) Schematic of the differentiation conditions used for the generation of in vitro NMPs from mouse pluripotent stem cells.

(B) Hierarchical clustering of all in vitro derived D3 cells using the genes contained in the three modules (identified from the in vivo population) reveals three distinct clusters. An e8.5 NMP identity characterized by genes in module 1, an e9.5 NMP identity characterized by genes in module 2, and a mesodermal characterized by genes in module 3.

(C) More than 60% of D3 NMP cells had a profile similar to e8.5 NMP cells, and 10% had a profile similar to e9.5 NMP cells.

(D) tSNE projection of e8.5, e9.5 and D3 NMPs using the genes comprising the three modules reveals that D3 cells appear more similar to e8.5 NMPs than e9.5 NMPs.

(E) Pseudotemporal ordering of in vitro generated NMP cells at D3 identifies a similar developmental trajectory to in vivo derived cells. The cell state graphs were obtained using the 99-gene signature of e-NMP cells partially shown in Figure 1F. The white-to-red colors indicate NMP-to-mesodermal fractional identity of each cell.

developmental trajectory. For example, *T/Bra*, *Nkx1.2*, *Cdx1, 2, 4*, and *Sox2* typical of e-NMPs, were expressed in cells at the initial stages of the pathway. *Sox2*, *Cdx2*, *Cdx4*, and *Nkx1.2* were downregulated as *Msgn1* and *Tbx6* were upregulated, whereas *Cdx1* expression was maintained (Deschamps and van Nes, 2005), which is characteristic of the transition from NMPs to MPCs (Figures 2E and S1A). This suggests that in vitro, as in vivo, the cells are following a similar developmental pathway. Taken together therefore, the data indicate that in vitro derived D3 NMPs resemble in vivo e-NMPs and display a similar developmental timeline.

Bifurcating Neural and Mesodermal Trajectories from NMPs

The in vivo and in vitro developmental trajectories showed the transition of NMPs to PSM via an MPC state, but in neither case was an obvious neural lineage observed. This was expected from the experimental conditions. In vivo, the dissection avoided cells in the neural region of the embryo, whereas the Wnt signaling used in vitro favors the mesoderm lineage. To extend our analysis to neural differentiation, we allowed in vitro NMPs to differentiate to neural progenitors by removing the Wnt signaling agonist at D3 and assaying cells at D4 in neurobasal (NB) medium containing vitamin A (the RA precursor) in the absence of Wnt signaling agonists (Figure 3A). Immunofluorescence analysis of T/Bra, Sox2, and Tbx6 at D4 indicated that, under these conditions, most cells acquired a neural identity while a small proportion continued to differentiate to mesoderm (Tbx6^{+}) (Figures 3B and 3C). We therefore analyzed the transcriptome of single cells at D3 and D4, excluding the cells that were expressing mature mesoderm markers such as *Meox1*. Hierarchical clustering of D3 and D4 single cells (using genes comprising the first four modules obtained using the same approach as applied to e-NMPs, Table S3) separated cells into four distinct groups. These clusters appeared similar to e8.5 NMPs, NMPs transitioning to a developmentally later (e9.0) NMP expression profile (t-NMP), PSM, and neural progenitor cells (NPCs) (Figure 3E).

Combining the gene expression data from D3 and D4 we constructed pseudotemporal orderings. Two distinct routes could be clearly distinguished from this analysis (Figure 3D). Examination of the genes differentially expressed along these routes indicated that they corresponded to mesodermal and neural developmental trajectories. The mesodermal route is characterized by the transient MPCs population expressing *T/Bra*, *Tbx6*, *Msgn1*, and *Nkx1.2*, as described above. By contrast, the neural lineage culminated in a population of cells that expressed *Sox1*, *Irx3*, *Zic1*, and *Zic2*, typical of NPCs. Along this neural trajectory, genes such as *T/Bra*, *E-Cadherin* (*Cdh1*), and *Pou5f1* (*Oct3/4*) associated with e8.5 NMPs were downregulated (Figures 3D and S1B).

We performed differential expression analysis of NPCs with NMPs to define genes associated with the neural trajectory. This analysis identified 143 genes differentially expressed. Out of these, 116 genes were downregulated as NMPs differentiated to neural progenitor cells, including *Cdx1*, *T/Bra*, *Wnt3a*, *Fst*, and *Cdh1*. The other 27 were induced as cells adopted a neural fate. These included *Sox1*, *Irx3*, but also *Meis1* and *Meis2*, which have previously been described to be expressed in neural tissue (Oulad-Abdelghani et al., 1997). Thus, this set of genes provides a molecular signature of neural induction from NMPs (Table S4).

Induction of NMPs Requires RA Signaling

Examination of the neural and mesodermal differentiation trajectories highlighted changes in the expression of RA signaling pathway components (Figure 3D). RA signaling is mediated by three RA receptors (RARα, β, and γ) that form heterodimers

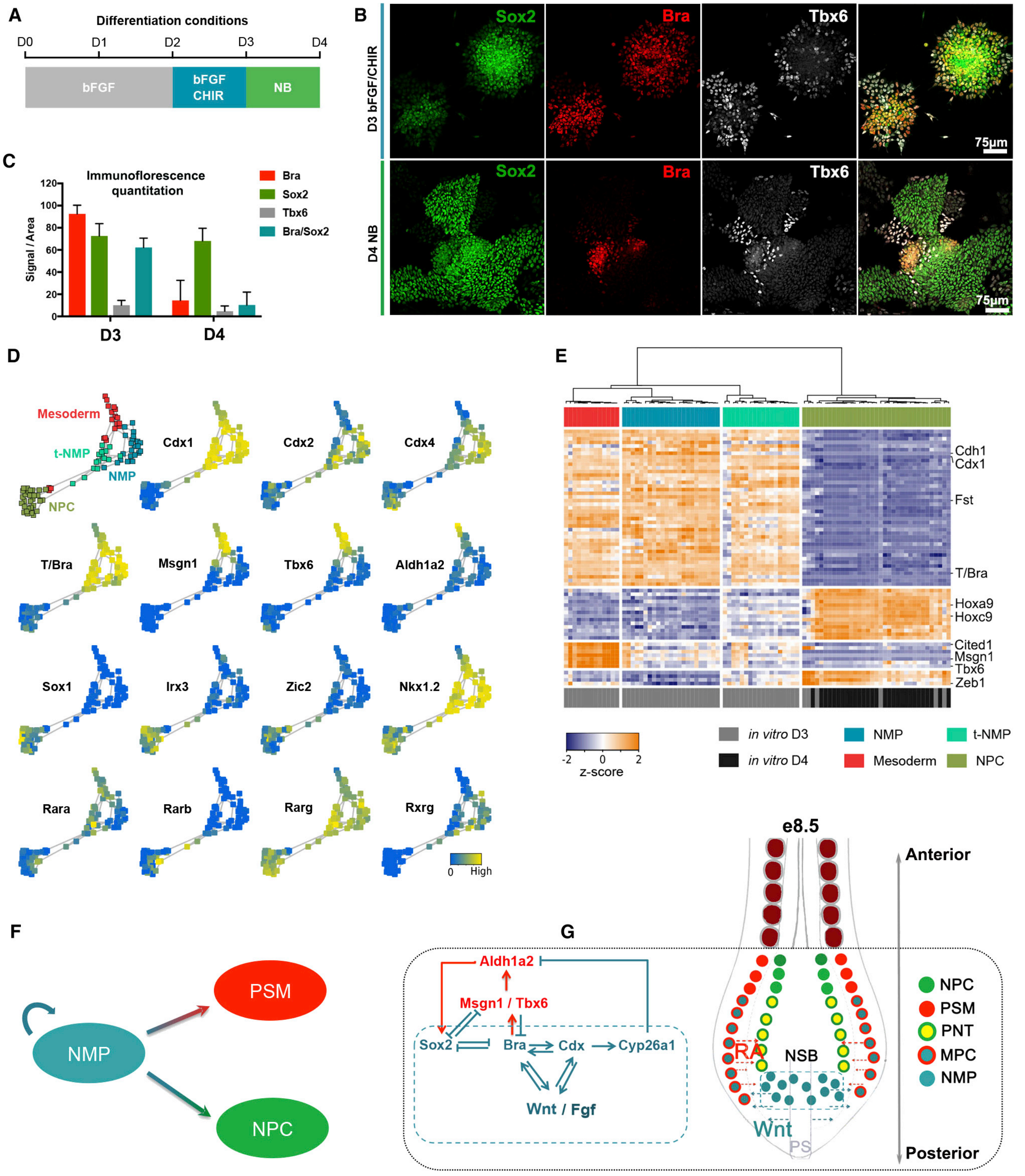

Figure 3. Single-Cell Analysis of the Differentiation Route of NMPs toward the Neural Lineage

(A) Schematic of the differentiation conditions used for the generation of in vitro NMPs and neural progenitor cells (NPCs) from mESCs.

(B) Immunohistochemistry of cultures at D3 indicates that most cells co-express Brachyury (T/Bra) and Sox2, characteristic of NMPs, and a small percentage of cells expressed Tbx6. Removal of CHIR after D3 and culture until D4 in NB (neurobasal) conditions induces the generation of NPCs that express Sox2 in the absence of T/Bra, and a few mesodermal cells expressing Tbx6.

(C) Quantitation of T/Bra^{+}, Sox2^{+}, Tbx6^{+}, or T/Bra^{+}/Sox2^{+} signal^{+} area normalized to DAPI area at D3 (bFGF/CHIR) and D4 (NB) of differentiation. Error bars indicate SD of four randomly selected independent fields.

(legend continued on next page)

with the retinoid X receptors (RXRα, β, and γ). In addition, aldehyde dehydrogenases (Aldh1a1, 2, and 3) play key roles in the synthesis of all-*trans*-RA from vitamin A. Consistent with the known in vivo expression profiles and functions (Ribes et al., 2009; Niederreither et al., 1997; Ang and Duester, 1997), the expression of nuclear RA receptor *RARβ* was upregulated in NPCs, whereas *RARα* was increased during PSM differentiation. *RARγ* and its binding partner *RXRγ* were expressed in NMP cells. Moreover, the expression of *Aldh1a2*, the rate-limiting enzyme for RA synthesis, correlated strongly with the expression of *Msgn1* and *Tbx6* (Figure 3D).

To test the possibility that RA functions during NMP differentiation, we abolished all RA signaling by generating an *Aldh1a2* mutant ESC line (*Aldh1a2*$^{-/-}$) and culturing these cells in the absence of vitamin A (STAR Methods). Strikingly, we found that removal of all RA signaling affected the ability to form T/Bra^{+}/Sox2^{+} NMP cells (Figures 4B, 4D, and S3B). *Sox2* expression was unaffected at D2, the time point at which differentiating ESCs display an epiblast-like identity (Figure S3B). Nevertheless, in *Aldh1a2*$^{-/-}$ cells, cultured in the absence of vitamin A, *Sox2* was downregulated at both the transcript and protein level at D3 (Figures 4B, 4D, 4J, and S3B). By contrast, the expression of the MPC markers *T/Bra*, *Msgn1*, and *Tbx6* was induced at higher levels than controls, suggesting that the cells more efficiently adopted a mesodermal identity in the absence of RA (Figure 4D). Strikingly, the expression of the early primitive streak/mesodermal markers *Eomes* and *Mixl1* was induced in the absence of RA (Figure S2A). These data suggest that in wild-type (WT) cells, low levels of RA are required for the induction of NMPs, whereas complete elimination of RA signaling leads to an anterior mesodermal identity (Figure 4A) (Kessel, 1992).

Previous studies in chick and mouse embryos have shown that premature exposure of the tail bud to high RA levels leads to axis truncation (Tenin et al., 2010; Olivera-Martinez et al., 2012). We tested whether this was also the case in vitro. Exposure of cells to a combination of bFGF/CHIR/RA (10 nM/100 nM) from D2 to D3 resulted in the downregulation of *T/Bra* and *Tbx6*, and the acquisition of a pre-neural tube (PNT) identity characterized by the expression of *Sox2*, *Sox1*, and *Nkx1.2* (Figures 4A, 4C, 4D, 4J, and S2A). These data agree with in vivo data in which RA treatment of e8.5 mouse embryos rapidly downregulates Wnt3a and T/Bra in the primitive streak (Figure 4D) (Shum et al., 1999; Iulianella et al., 1999).

Collectively, the data identify a novel role for RA in NMPs. In the absence of RA, Wnt/FGF signaling promotes an anterior mesodermal identity (Bra^{+}/Tbx6^{+}/Cdx^{-}), and low levels of RA are necessary for the induction of NMP identity (Bra^{+}/Sox2^{+}), whereas high levels of RA produce pre-neural progenitors (Sox2^{+}/Nkx1.2^{+}). This suggests that tightly controlled levels of RA signaling are necessary for the induction of NMP and neural identity (Figure 4A, model).

Cdx Genes Maintain the NMP State by Suppressing RA Signaling

The failure to induce NMP cells in the absence of RA was accompanied by a downregulation of *Cdx* expression (Figure S2B). This prompted us to address the role of *Cdx* genes in NMPs. Previous studies have implicated the *Cdx* genes in the induction of *T/Bra*, *Wnt3a*, *Fgf8*, posterior *Hox* genes, and the RA-degrading enzyme *Cyp26a1*. However the presence of three functionally similar *Cdx* genes (*Cdx1, Cdx2,* and *Cdx4*) and the failure of axis elongation when all three are removed has complicated in vivo analysis and made it difficult to define a regulatory hierarchy.

We constructed ESCs harboring nonsense mutations in all six alleles of the *Cdx* genes (STAR Methods). We designated these *Cdx*$^{1,2,4-/-}$ cells. We then differentiated *Cdx*$^{1,2,4-/-}$ ESCs cells using NMP-inducing conditions (Gouti et al., 2014) (Figure 4E). Consistent with the established role of *Cdx* in the activation of posterior *Hox* genes, there was an absence of posterior *Hox* gene expression in *Cdx*$^{1,2,4-/-}$ cells, although the expression of anterior *Hox* genes was maintained (Figure S3G) (Mazzoni et al., 2013; Neijts et al., 2016).

At D3 *T/Bra* was expressed in *Cdx*$^{1,2,4-/-}$ cells, albeit it at lower levels compared with controls (Figures 4F, 4I, and 4J). *Tbx6* was induced in *Cdx*$^{1,2,4-/-}$ cells at similar levels, whereas the expression of *Msgn1* was lower compared with control cells at D3 (Figures 4F, 4I, 4J, and S3D). The expression of T/Bra was downregulated at D4 in the *Cdx*$^{1,2,4-/-}$ cells even in the presence of Wnt signaling (Figures S3C and S3D). This suggests that, although *Cdx* activity is not necessary to initiate *T/Bra* and *Tbx6* expression, they act with Wnt/FGF signaling to maintain *T/Bra* and enhance *Msgn1* expression. Consistent with the early downregulation of *T/Bra*, *Tbx6* expression was not maintained in *Cdx*$^{1,2,4-/-}$ cells cultured under prolonged Wnt conditions (5 μM CHIR until D5) (Figures 4G, 4J, and 4I), and the cells acquired a

(D) Pseudotemporal ordering of D3 and D4 cells identifies four different populations and two distinct differentiation trajectories that lead to either neural or mesodermal identity. Expression of *Cdx1*, *Cdx2*, *Cdx4*, *T/Bra*, and *Nkx1.2* is high in NMP cells, the expression of mesoderm specific genes *Msgn1* and *Tbx6* is high in Mesoderm cells. By contrast, there is induction of *Sox1*, *Irx3*, and *Zic2* along the neural trajectory. The expression of RA signaling pathway components is differentially regulated in each developmental trajectory. *RXRγ* and *RARγ* are co-expressed in D3 NMP cells (similar to e8.5 NMPs). Expression of *Aldh1a2* correlated strongly with *Msgn1* and *Tbx6* expression. See also Figure S1.

(E) Hierarchical clustering of D3 and D4 single cells partitions the cells into four major groups. An NMP cluster (NMP, cerulean), a transitioning NMP (t-NMP, cyan) that express markers of developmentally older NMPs, a mesodermal (Meso, red), and a neural progenitor cell cluster (NPC, green). D3 cells are indicated in gray and D4 cells in black. See Tables S3 and S4.

(F) Diagram illustrating the mesoderm or neural progenitor fate choice made by NMP cells.

(G) Schematic of the posterior part of an e8.5 mouse embryo. NMP cells (cerulean) expressing *Bra/Sox2/Cdx* genes are located in the CLE region, close to the NSB in the anterior part of the primitive streak. As cells leave the NMP zone they differentiate to MPC progenitors (cerulean/red) expressing *Bra/Msgn1/Tbx6*, which results in upregulation of *Aldh1a2*. Thus, increased levels of RA produced in close proximity to the niche promote *Sox2* expression and the differentiation of NMPs to PNT cells (green/yellow) then NPCs (green). The transcriptional network that controls the cell fate decision of NMP cells toward neural or mesodermal identities is summarized adjacent to the embryo model. NMP, neuromesodermal progenitor; t-NMP, transitioning neuromesodermal progenitor; NPCs, neural progenitor cells; PSM, presomitic mesoderm; MPC, mesodermal progenitor cells; PNT, pre-neural tube cells; NSB, node-streak border; PS, primitive streak; NB, neurobasal conditions.

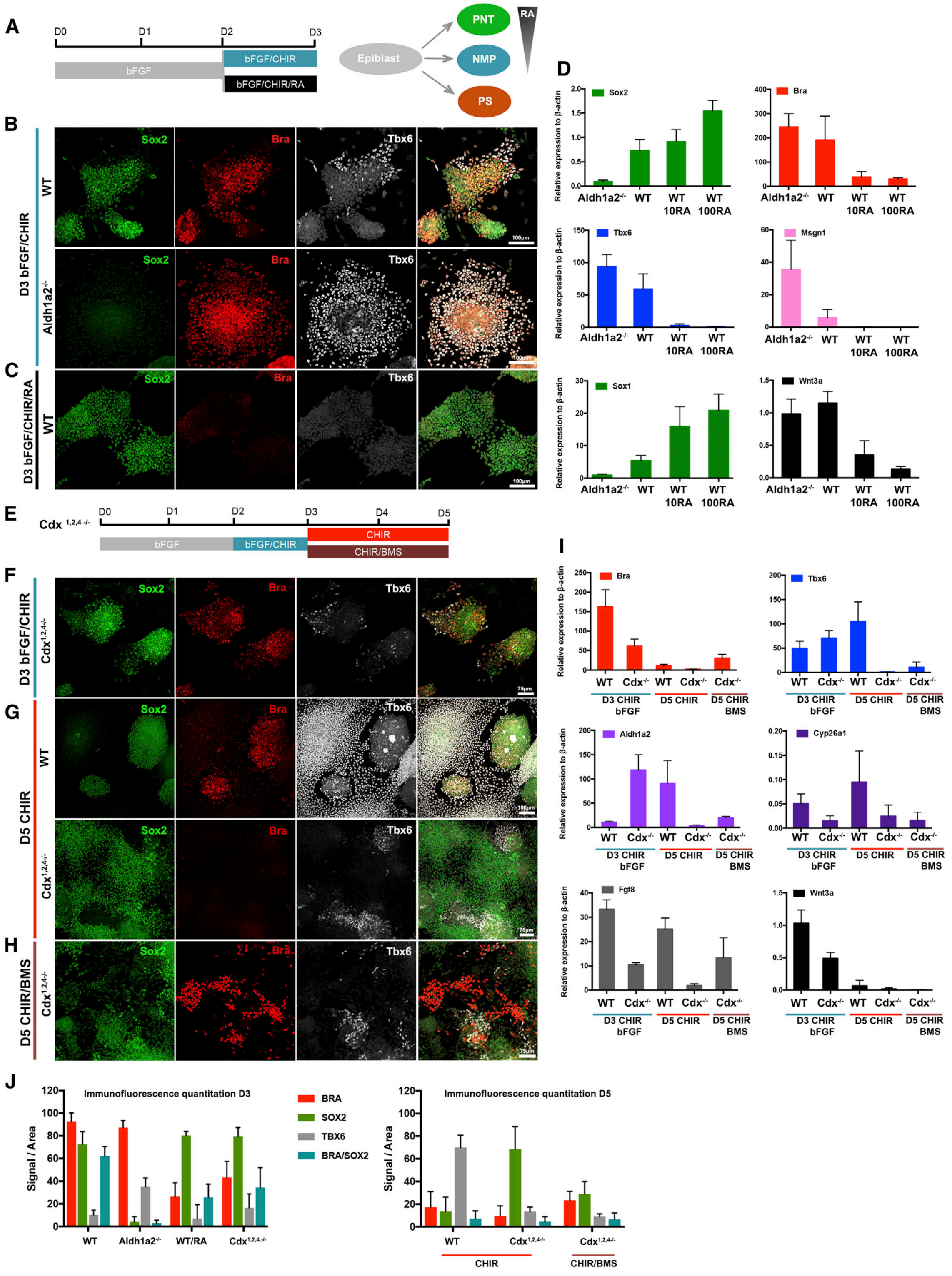

Figure 4. Induction of NMPs Requires Low Levels of RA Signaling

(A) Schematic of in vitro differentiation conditions and diagram illustrating the different cell fate choices of epiblast cells in the presence of bFGF/CHIR signaling and variable levels of RA.

(B) Immunohistochemistry at D3 of differentiation indicates that *Aldh1a2*$^{-/-}$ ESCs exposed to bFGF/CHIR downregulate Sox2 and express the mesodermal markers T/Bra and Tbx6. WT ESCs differentiated under the same conditions co-express T/Bra and Sox2.

(legend continued on next page)

neural fate characterized by the expression of Sox2, Sox1, and Tuj1 (Figures 4G, 4J, S3E, and S3F). In contrast, WT cells under the same conditions adopted predominantly a PSM identity, characterized by the expression of Tbx6 and the downregulation of T/Bra (Figures 4G and 4I).

The expression of *Wnt3a* and *Fgf8* was significantly downregulated in *Cdx*$^{1,2,4-/-}$ cells (Figure 4I), consistent with previous reports (Savory et al., 2009). However, since the exogenous supplementation of Wnt/Fgf is not sufficient to rescue the differentiation defects in *Cdx*$^{1,2,4-/-}$ cells, there must be additional targets of Cdx function. Inspection of the developmental timeline revealed an inverse relationship between the expression of the *Cdx2*, *Cdx4*, and RA production (Figure 3D). Consistent with this, expression of *Aldh1a2* was strongly induced in *Cdx*$^{1,2,4-/-}$ cells, whereas the expression of *Cyp26a1* was significantly downregulated at D3 under NMP conditions (Figure 4I). The increased expression of *Aldh1a2* in *Cdx*$^{1,2,4-/-}$ cells, together with the evidence that high levels of RA antagonize NMPs prompted us to ask whether increased production of RA in *Cdx*$^{1,2,4-/-}$ cells promotes abnormal neural differentiation. To test this, we cultured D3 *Cdx*$^{1,2,4-/-}$ in NMP-inducing conditions and exposed these to an RA inhibitor (1 μM BMS) from D3 to D5 (Figure 4E). This resulted in the induction of a mesodermal population in the *Cdx*$^{1,2,4-/-}$cells that expressed T/Bra (Figures 4H and 4J). However, Sox2 was downregulated, indicating the absence of NMPs (Figures 4H and 4J). Thus, in the absence of *Cdx*$^{1,2,4-/-}$, mesoderm induction can be partially restored by the inhibition of RA signaling, suggesting an important role for *Cdx* in controlling levels of RA signaling to allow mesoderm and NMP production.

Taken together these data show that *Cdx* genes are necessary in establishing NMP identity and subsequently for the differentiation of spinal cord and PSM cells. This is achieved by inducing the expression of posterior *Hox* genes and by regulating the balance of Wnt3a, Fgf8, and RA signaling.

Msgn1 Promotes the MPC-to-PSM Transition

The production of neural cells in the absence of *Cdx* activity under continuous Wnt signaling led us to focus on the mechanisms that drive the exit from NMP identity. During the differentiation of NMPs to mesoderm, cells transit through an MPC state that co-expresses *T/Bra*, *Msgn1*, and *Tbx6* and then downregulate *T/Bra* adopting a PSM identity (*Msgn1*$^+$*Tbx6*$^+$) (Chalamalasetty et al., 2014) (Figures 1D and 2E). Mutations in *Msgn1* or *Tbx6* in vivo affect the production of PSM tissue causing defects in axis elongation (Chapman et al., 2003; Chapman and Papaioannou, 1998; Yoon and Wold, 2000).

To test how *Msgn1* affects PSM differentiation and what role it plays in the exit from the NMP state, we generated a *Msgn1* mutant ESC line (*Msgn1*$^{-/-}$) (STAR Methods). We induced the differentiation of *Msgn1*$^{-/-}$ ESCs to NMPs (Figure 5A). At D3, NMPs were generated with high efficiency, demonstrated by the co-expression of T/Bra and Sox2 (Figures 5B and 5E). Notably, expression of *T/Bra* was consistently higher in the *Msgn1*$^{-/-}$ NMPs compared with the WT cells (Figure 5F). We then exposed NMPs to continuous Wnt signaling (CHIR) to promote differentiation into PSM. In contrast to WT cells, which have downregulated T/Bra and express the PSM markers Tbx6 by D5, *Msgn1*$^{-/-}$ ESCs maintained the expression of *T/Bra*, *Sox2*, *Nkx1.2*, and low levels of *Tbx6* (Figures 5C, 5E, and 5F). Thus, in the absence of *Msgn1*, cells are unable to progress to PSM and appear to be trapped in an NMP/MPC stage of differentiation. The consistently higher levels of *T/Bra* in the *Msgn1*$^{-/-}$ cells imply that *Msgn1* has a negative feedback on *T/Bra* to allow the exit from the MPC state. This is consistent with the in vivo data and suggests that *Msgn1* acts specifically to promote the MPC to PSM transition (Chalamalasetty et al., 2014; Yoon and Wold, 2000).

We next asked whether the absence of *Msgn1* affected neural differentiation. Surprisingly, although the withdrawal of the Wnt agonist allowed the neural differentiation of *Msgn1*$^{-/-}$ ESCs, this was substantially delayed compared with WT cells. In the *Msgn1*$^{-/-}$ESCs the expression of T/Bra was maintained longer even under NB conditions, and there was a ~24 hr delay in the induction of neural identity compared with controls (Figures 5D and S4). Since *Msgn1* expression is largely confined to cells that adopt a PSM fate, these data suggested that *Msgn1* induces the production of a signal that acts non-autonomously to promote neural differentiation and block T/*Bra* expression. Examination of the single-cell transcriptome data revealed a strong correlation between *Msgn1* expression and *Aldh1a2*, the RA synthesis enzyme (Figure 3D). We hypothesized that the delay in neural differentiation in the absence of *Msgn1* might be due to low levels of *Aldh1a2*. To test this, we increased RA signaling

(C) Exposure of cells to increased levels of RA from D2 to D3 eliminates NMP induction and instead induces an NPC identity, evident by the expression of Sox2 in the absence of T/Bra and Tbx6.

(D) qRT-PCR analysis of the expression of *Sox2*, *T/Bra*, *Tbx6*, *Msgn1*, *Sox1*, and *Wnt3a* at D3 in *Aldh1a2*$^{-/-}$cells and WT ESCs treated with bFGF/CHIR or bFGF/CHIR/RA (RA 10 or 100 nM). Mesodermal markers are induced in *Aldh1a2*$^{-/-}$ cells, whereas the expression of *Sox2* and *Sox1* is abolished. By contrast, increasing RA concentrations induce neural fate identity, characterized by the upregulation of *Sox2* and *Sox1*, whereas the expression of the mesodermal genes, *T/Bra*, *Msgn1*, and *Tbx6* is absent. Expression of *Wnt3a* is significantly dowregulated under RA conditions. See also Figure S2.

(E) Schematic of differentiation conditions used for *Cdx*$^{1,2,4-/-}$cells.

(F) Immunohistochemistry at D3 indicates the induction of cells that co-express T/Bra and Sox2. Also, Tbx6 expression is initially induced in the *Cdx*$^{1,2,4-/-}$cells.

(G) Although continuing exposure to CHIR results in WT cells predominantly adopting Tbx6-expressing mesodermal identity, *Cdx*$^{1,2,4-/-}$cells acquire a Sox2-expressing NPC identity.

(H) Inhibition of RA signaling (with 1 μM BMS) partially restores mesodermal differentiation, revealed by the upregulation of T/Bra in the *Cdx*$^{1,2,4-/-}$ cells.

(I) qRT-PCR analysis of mesodermal genes *T/Bra* and *Tbx6*, RA signaling pathway components *Cyp26a1*, *Aldh1a2,* and Wnt and Fgf signaling ligands *Wnt3a* and *Fgf8* in *Cdx*$^{1,2,4-/-}$ and WT cells at D3 (NMP conditions), D5 (CHIR conditions), or D5 CHIR conditions with RA inhibition (1 μM BMS) from D3 to D5. In the *Cdx*$^{1,2,4-/-}$cells the expression of *T/Bra* is induced at D3 but at lower levels, and the expression of *Aldh1a2* is substantially increased compared with WT ESCs. At D5, expression of mesodermal markers *T/Bra* and *Tbx6*, as well as *Wnt3a* and *Fgf8*, is downregulated in *Cdx*$^{1,2,4-/-}$cells. RA inhibition in the presence of Fgf signaling results in partial restoration of *T/Bra* and *Fgf8* in the *Cdx*$^{1,2,4-/-}$cells. qRT-PCR data were normalized against *β-actin*. Error bars indicate SD of three biological replicates. See also Figure S3.

(J) Quantitation of Bra$^+$, Sox2$^+$, Tbx6$^+$, or Bra$^+$/Sox2$^+$ signal$^+$ area normalized to DAPI area at D3 (bFGF/CHIR) and D5 (CHIR or CHIR/BMS) of differentiation. Error bars indicate SD of four randomly selected independent fields. PNT, pre-neural tube; PS, primitive streak.

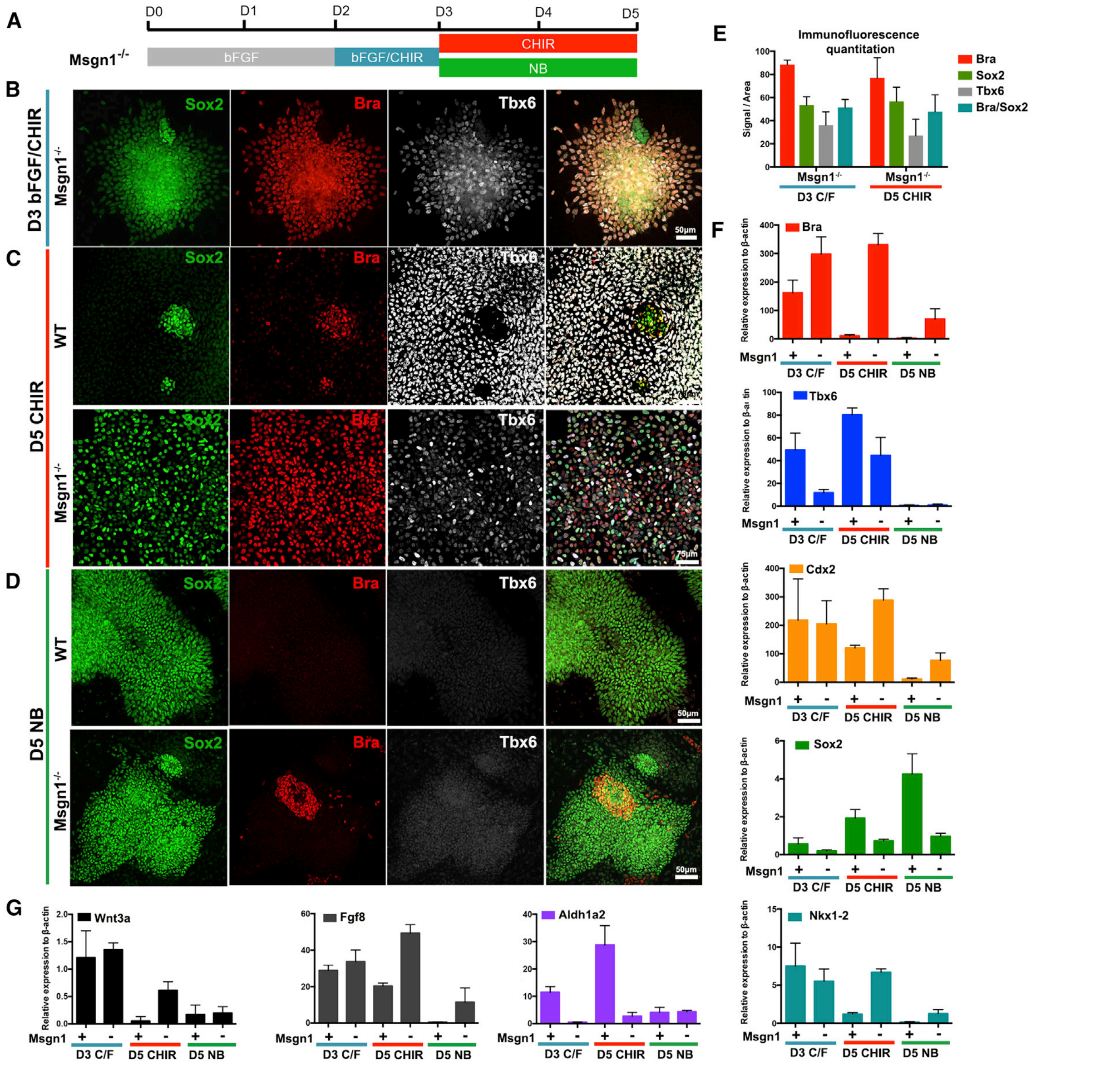

Figure 5. NMP Cells Are Generated in the Absence of *Msgn1* but Cannot Efficiently Differentiate to PSM Identity

(A) Schematic of in vitro culture conditions used for assaying *Msgn1*$^{-/-}$ cells.

(B) Immunohistochemistry of *Msgn1*$^{-/-}$ cells at D3 of differentiation reveals most cells co-express T/Bra and Sox2. The expression of Tbx6 is also evident in some cells at D3.

(C) At D5 under mesodermal conditions WT ESCs predominantly differentiate to PSM, whereas *Msgn1*$^{-/-}$ cells are maintained in an NMP state co-expressing T/Bra with Sox2. Tbx6 is also expressed in some cells, but these have not downregulated T/Bra.

(D) Downregulation of T/Bra is delayed in the *Msgn1*$^{-/-}$ cells under NB conditions, as is evident by the presence of T/Bra-expressing cells at D5. See also Figure S4.

(E) Quantitation of T/Bra^{+}, Sox2^{+}, Tbx6^{+}, or T/Bra^{+}/Sox2^{+} signal^{+} area normalized to DAPI area at D3 (bFGF/CHIR) and D5 (CHIR) of *Msgn1*$^{-/-}$ ESC differentiation. Error bars indicate SD of four randomly selected independent fields.

(F) qRT-PCR analysis of *T/Bra*, *Tbx6*, *Cdx2*, *Sox2* and *Nkx1.2* at D3 (NMP conditions), D5-CHIR (mesodermal conditions) and D5 NB (neural conditions) in *Msgn1*$^{-/-}$ and WT cells. At D3 *Msgn1*$^{-/-}$ cells express high levels of *T/Bra* and lower levels of *Tbx6* compared with controls. *Cdx2* and *Nkx1.2* expression is not affected, whereas *Sox2* is downregulated. At D5 CHIR conditions *Msgn1*$^{-/-}$ cells express high levels of NMP markers, *T/Bra*, *Sox2*, *Cdx2*, *Nkx1.2* and lower levels of *Tbx6*, compared with WT cells that have acquired a PSM identity.

(G) The expression of *Wnt3a* and *Fgf8* is significantly higher in the *Msgn1*$^{-/-}$ cells at D5 CHIR, whereas the expression of *Aldh1a2* is downregulated. qRT-PCR data were normalized relative to *β-actin*. Error bars indicate SD of three biological replicates.

by supplementing the medium with 100nM RA from D3 to D5 (Figure S4). This restored the timely downregulation of T/Bra and the upregulation of neural markers.

Taken together, the data indicate that *Msgn1* is required to drive the cells from an MPC to a PSM state. This is achieved in part by the cell-autonomous enhancement of mesoderm

differentiation. In addition, *Msgn1*-mediated upregulation of *Aldh1a2* increases RA synthesis, promoting the non-autonomous repression of T/Bra and neural differentiation (Iulianella et al., 1999; Martin and Kimelman, 2010; Sakai et al., 2001).

RA Signaling Regulates the Exit from the NMP State in Concert with Tbx6

Since *Tbx6* is induced simultaneously with *Msgn1* and is implicated in the induction of PSM (Chapman and Papaioannou, 1998; Takemoto et al., 2011; Bouldin et al., 2015; Nowotschin et al., 2012) (Figures 1D and 2D), we compared the role of *Tbx6* with that of *Msgn1*. To this end we used a previously published *Tbx6* mutant (*Tbx6*$^{-/-}$) ESC line (Chapman et al., 2003).

Differentiation of *Tbx6*$^{-/-}$ ESCs to NMPs was efficient, with most cells co-expressing T/Bra and Sox2 at D3 (Figure 6B). Continued Wnt signaling (CHIR) resulted in *Tbx6*$^{-/-}$ cells remaining in a T/Bra^{+}/Sox2^{+} state at D5 (Figure 6C). Expression of *Msgn1* was transiently present at D3, but was not maintained, consistent with the requirement in vivo for Tbx6 to maintain Msgn1 (Chalamalasetty et al., 2011) (Figure 6E). Moreover, the expression of *Wnt3a*, *Fgf8*, *Nkx1.2* and *Cdx2* was maintained at D5 in *Tbx6*$^{-/-}$ cells, whereas the expression of *Aldh1a2* was low (Figures 6E). This suggested that Tbx6-mediated induction of *Aldh1a2* under continuous Wnt signaling drives the exit toward the neural lineage. Thus, in the absence of *Tbx6*, NMP cells can be maintained under prolonged Wnt conditions.

To investigate how long the NMP state could be maintained we passaged *Tbx6*$^{-/-}$cells under bFGF/CHIR conditions and assayed gene expression. By D7 WT cells had adopted a mixed PSM and neural identity (Figure 6F). By contrast, *Tbx6*$^{-/-}$ cells continued to co-express T/Bra and Sox2 (Figure 6F). However, after the second passage many *Tbx6*$^{-/-}$ cells had acquired a neural identity, suggesting that continued Wnt/Fgf signaling is insufficient to block neural differentiation. We hypothesized that low levels of RA in these culture conditions might allow neural differentiation. To test this we removed vitamin A from the culture medium after D3. Under these conditions *Tbx6*$^{-/-}$ cells could be maintained in an NMP state for up to 10 days (three passages), albeit it with a declining growth rate over time (Figure 6F). These data are consistent with the idea that, although RA initially induces NMP identity, subsequently RA signaling or the induction of *Tbx6* promotes exit from the NMP state.

We then compared *Hox* gene expression in NMPs. At D3, NMPs expressed 3′ Hox genes such as *Hoxb1*. Strikingly, at D5, most *Tbx6*$^{-/-}$ cells co-expressed T/Bra with Hoxc10 (Figure 6D), normally associated with lumbar spinal cord, suggesting an acquisition of NMPs with more posterior axial identity (similar to e9.5 NMPs). Moreover, analysis of the *Hox* profile of *Tbx6*$^{-/-}$ NMPs at D9 revealed activation of the *Hox13* paralog (Figure 6G). This suggests that in vitro, as in vivo, the axial identity of NMP cells progressively changes with time. To systematically test the similarity between the in vivo and in vitro gene expression program associated with the axial identity of NMPs, we performed single-cell sequencing on in vitro derived *Tbx6*$^{-/-}$ mutant NMPs at D3 and D6. A tSNE projection, using the 168 genes with the highest dispersion among the samples (Satija et al., 2015), indicated that D3 NMPs were most similar to e8.5 NMPs, whereas D6 NMPs mapped closest to e9.5 NMPs (Figure 6H). Moreover examining the genes identified as changing with embryonic time in vivo confirmed similar changes of gene expression in vitro (Figures 6H–6J). Together these data establish the similarity in behavior between in vitro and in vivo NMPs and define a temporal program that characterizes progressive changes in NMP gene expression. Hence, the temporal changes in axial identity of NMPs is also recapitulated in vitro.

A Dynamical Model of NMP Induction and Differentiation

The identification of a role for RA in NMP induction and the availability of neural and mesodermal developmental timelines prompted us to explore whether this was sufficient to reverse engineer the underlying gene regulatory network. To this end, we constructed a dynamical model of linked ordinary differential equations describing gene activity. To identify relevant topologies and parameterize these models we took advantage of the gene expression dynamics revealed by the pseudotemporal orderings of single cells (Figure 3) and the results from the genetic perturbations (Figures 4, 5, and 6).

To reconstruct a minimal network we restricted our analysis to the regulatory relationships among the four core TFs *T/Bra*, *Sox2*, *Msgn1*, and *Tbx6*. Because of the tightly correlated expression and activity of *Msgn1* and *Tbx6*, we used a single node to represent both genes. In the resulting network, we allowed all possible interactions between the three TF nodes (T/*Bra*, *Sox2*, and *Msgn1/Tbx6*). In total, this gave six possible interactions between the three TFs, and each could act as an activator or repressor, leading to 64 topologies (Figure 7B). In addition, the *T/Bra* and *Sox2* nodes were connected to the two upstream signals Wnt/Fgf and RA (Figure 7C). For each topology, we identified best-fit parameters by running parallel differential evolution optimizations and comparing the resulting simulated dynamics of gene expression with a set of objectives. These objectives represented the gene expression trajectories from D2 of differentiation to *Tbx6*$^{+}$/*Msgn1*$^{+}$ expressing mesoderm in the presence of Wnt/Fgf (Meso), or to *Sox2*$^{+}$/*Bra*$^{-}$ neural progenitors in the presence of RA (neural). In addition, we included conditions representing *T/Bra* and *Tbx6/Msgn1* mutants and conditions in which in vitro NMPs are not produced (absence of Wnt signaling from D2 to D3) (Figure 7A).

The optimizations identified two topologies that generated good fits (Figure 7C). In both of these, all three TFs cross-repress each other, with the exception of the interaction of *T/Bra* on *Tbx6*, which could be either activating or weakly repressing. Examination of these topologies suggested an underlying logic in which, a toggle switch-like mechanism between a *Sox2*^{HIGH}/*Tbx6*^{LOW} (neural) state and a *Sox2*^{LOW}/*Tbx6*^{HIGH} (mesoderm) state is coupled to regulative feedback provided by RA. In this way, the circuit enforces a decision between neural and mesodermal states, with the feedback ensuring the balanced production of the two cell types: *Tbx6* (mesoderm) increases RA and thereby increases *Sox2* (neural) production in surrounding cells, conversely, low levels of *Tbx6* increases *T/Bra* production and thereby *Tbx6* (mesoderm) production.

We reasoned that this mechanism, together with the inherent stochasticity of gene expression, could be sufficient to explain the coordinated generation of neural and mesodermal tissue from NMPs. To test this, we performed simulations with stochastic differential equations and examined the dynamical trajectory and the proportions of each state produced. Consistent with the

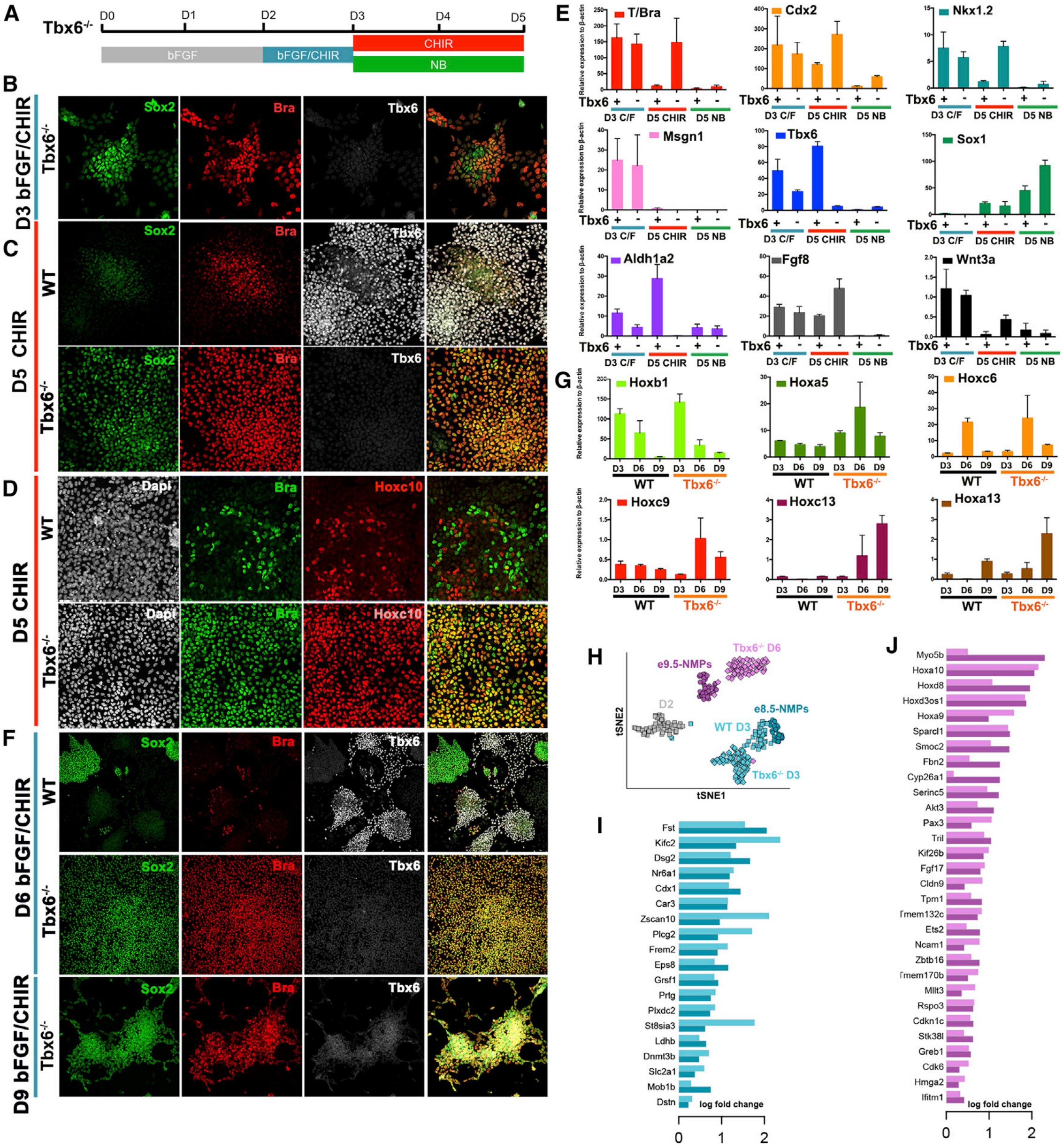

Figure 6. Maintenance of NMP Cell Identity in the Absence of Tbx6

(A) Schematic of conditions to assay *Tbx6*$^{-/-}$ ESCs.

(B) *Tbx6*$^{-/-}$ cells co-expressing T/Bra and Sox2 at D3 under NMP conditions (bFGF/CHIR).

(C) *Tbx6*$^{-/-}$ cells are maintained as NMPs characterized by the co-expression of T/Bra and Sox2 at D5 (CHIR conditions), whereas WT cells mostly downregulate T/Bra and Sox2 and instead express Tbx6.

(D) At D5 in CHIR conditions, *Tbx6*$^{-/-}$ cells acquire an identity more similar to e9.5 NMPs characterized by the co-expression of T/Bra^{+}/Sox2^{+} with Hoxc10^{+}. In WT cells, few late NMP cells could be detected, as most Hoxc10-expressing cells were T/Bra negative.

(E) qRT-PCR analysis of NMP, mesodermal, and neural markers at D3 (NMP conditions), D5 CHIR (mesodermal conditions), and D5 NB (neural conditions) shows that NMPs are induced at D3 in the *Tbx6*$^{-/-}$cells and maintained during exposure to CHIR. Expression of *Aldh1a2* is upregulated at D3 and D5 (CHIR) in WT ESCs, whereas *Tbx6*$^{-/-}$ cells express low levels of *Aldh1a2* and higher levels of *Fgf8* and *Wnt3a* at D5 (CHIR).

(F and G) The *Tbx6*$^{-/-}$ cells maintained NMP identity for 9 days (two passages) under bFGF/CHIR conditions (F) and progressively express more posterior *Hox* genes (G).

(H) tSNE projection of the in vitro WT and *Tbx6*$^{-/-}$ passaged NMPs with e8.5 and e9.5 NMPs revealed that D3 WT and *Tbx6*$^{-/-}$ in vitro NMPs are similar to e8.5 NMPs, whereas the passaged D6 *Tbx6*$^{-/-}$ in vitro NMPs closely resemble e9.5 NMPs.

(I and J) Taking the intersection of differentially expressed genes identified between in vitro D3 *Tbx6*$^{-/-}$ and D6 *Tbx6*$^{-/-}$ cells, and in vivo e8.5 and e9.5 NMPs showed similar changes in gene expression of early (I) and late NMP signature genes (J).

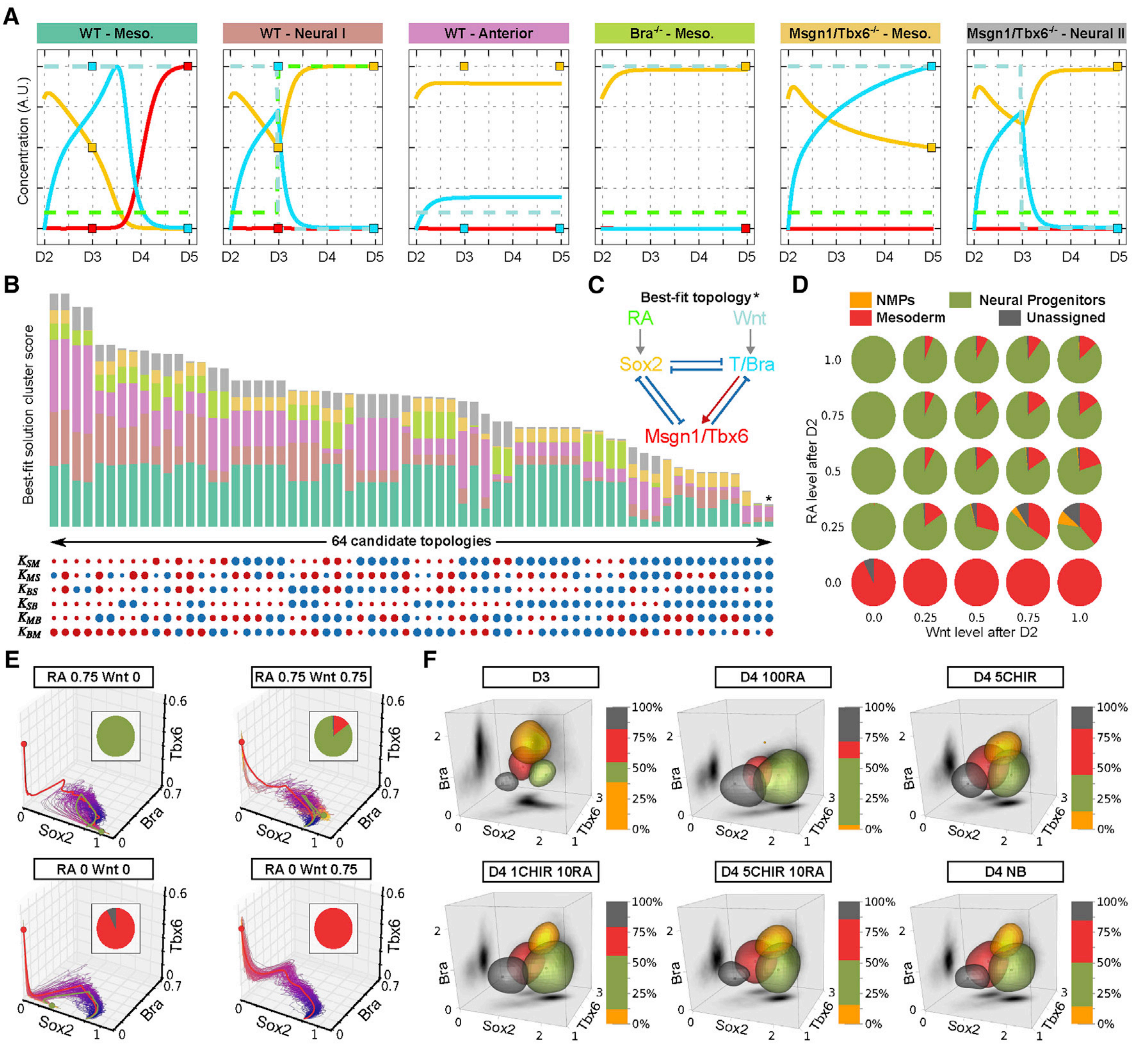

Figure 7. Reverse Engineering the NMP Gene Regulatory Network

(A) Six dynamical patterns summarize the experimental observations used as objectives to identify the best-fit network topology. Targeted time points are squares positioned at observed gene levels for *Sox2* (orange), *T/Bra* (blue), and *Msgn1/Tbx6* (red). The x axis represents the simulated time between D2 and D5. The y axis represents the protein concentration in a.u. All simulations are performed with a deterministic ordinary differential equation model from the initial conditions: *Sox2*^{HIGH}, T/*Bra*^{LOW}, and *Msgn1/Tbx6*^{LOW}. In addition to WT simulations, *T/Bra* and *Msgn1/Tbx6* mutant simulations were performed by setting the relevant protein production rate to zero. Dashed lines represents the four *RA/Wnt* signaling conditions and solid lines a typical best-fit solution.

(B) Overview of the 64 parallel parameter explorations evaluated. Each bar plot represents the average score of the best-fit solution cluster for one topology (a perfect score is 0). Bar colors identify the six objectives shown in (A). Underneath, circles describe the associated topology (blue circles for repression, red circles for activation) and the radius of each circle is proportional to the binding affinities in the best-fit solutions.

(C) The best-fit topology identified by the parameter exploration.

(D) Results of stochastic simulations. Colored pie charts show the ratios of cell states observed at D5 of stochastic simulations using the optimal topology under various *RA/Wnt* conditions between D3 and D5. Prior to D3, all trajectories are simulated with the same *RA*^{LOW}/*Wnt*^{HIGH} signaling condition.

(E) Stochastic trajectories obtained with four *RA/Wnt* signaling conditions that are representative of the four conditions in (D). The thick solid lines indicate the average trajectories for neural (green) and mesodermal (red) fates. Circles represent the attractors of the dynamical system.

(F) Experimental results. The ratios of different cell types obtained by co-measuring Sox2, T/Bra, and Tbx6 levels by flow cytometry. Assays were conducted using six *RA/Wnt* signaling conditions between D3 and D4. Grayscale panels show 2D kernel density estimations for each cell obtained from the protein fluorophore intensities. Contour plots document the highest density regions of the four cell populations identified by k-means clustering. Axes follow the "logicle" scale (Parks et al., 2006). Associated bar plots indicate the ratios of cell types in each condition.

deterministic model, in neural conditions the majority of stochastic simulations generated a *Sox2*^{HIGH}/*Tbx6*^{LOW} endpoint after transiently adopting a *Sox2*^{MED}/*Bra*^{HIGH}NMP-like state. Similarly, in mesoderm conditions an NMP-like state was transiently visited but now most stochastic simulations ended in a *Sox2*^{LOW}/*Tbx6*^{HIGH} (mesoderm) state (Figure 7E). We next performed a series of simulations in which we systematically varied the levels of RA and Wnt in order to generate an input/output (I/O) map. This map was consistent with the regulative role of Wnt and RA feedback. Increasing the level of Wnt increased the proportion of

cells that adopted a mesoderm identity, whereas RA biased cells to neural. Combinations of both signals produced mixtures of cell types (Figure 7D).

The simulations appeared consistent with the experimental data generated by manipulating Wnt and RA signaling levels (Figures 3 and 4). To examine this in more detail we used fluorescence-activated cell sorting to quantify the proportion of differentiating in vitro NMPs expressing Bra/Sox2/Tbx6 under defined conditions. We used the fluorescent intensity levels of the three assayed TFs to define four clusters. These correspond to NMP (Bra^{HIGH}/Sox2^{LOW}/Tbx6^{LOW}), neural (Bra^{LOW}/Sox2^{HIGH}/Tbx6^{LOW}), mesoderm (Bra^{LOW}/Sox2^{LOW}/Tbx6^{HIGH}), and unassigned (low for all three markers). We first examined NMPs cultured from D3 to D4 in NB conditions without RA or Wnt signaling. At D4 the number of cells in the NMP compartment had markedly reduced, and approximately equal proportions of neural and mesoderm cells were observed (Figure 7F). Exposing NMP cells to 100nM RA from D3 to D4 decreased the proportion of mesodermal cells substantially, concomitantly increasing the number of neural cells. Conversely, the mesoderm compartment increased at the expense of neural cells in NMPs exposed to 5μM CHIR from D3 to D4. Exposure of cells to a combination of 10nM RA and 5 μM CHIR resulted in a similar proportion of cell types to basal conditions. Moreover, maintaining 10nM RA but reducing the level of Wnt signaling to 1μM CHIR increased the proportion of neural cells and reduced mesoderm proportion (Figure 7F). Taken together these data are consistent with the I/O map and confirm the regulative activity of RA and Wnt signaling.

DISCUSSION

We provide a detailed molecular description of bipotential NMPs and reconstruct their differentiation into neural and mesodermal tissue. Single-cell transcriptomics define gene expression signatures characteristic of NMPs and provide evidence that in vitro and in vivo NMPs are molecularly and functionally similar. These data enabled the reverse engineering of the underlying gene regulatory network and highlighted a role for RA signaling in establishing and resolving the NMP state. This revealed a mechanism that regulates the proportion of neural and mesodermal cells generated and offered insight into the network architecture that facilitates orderly axis elongation and the elaboration of the mammalian trunk.

A Gene Expression Signature of NMPs

Microdissection of embryonic tissue followed by single-cell transcriptome assays established a gene expression signature shared by e8.5 and e9.5 NMPs as well as identifying the changes associated with embryonic age. As anticipated, the core NMP signature included *T/Bra*, *Nkx1.2*, *Sox2*, *Cdx2*, and *Sp8*. In addition, genes such as *Epha2*, *Epha5*, *Pou5f1* (*Oct3/4*), *Sema6a*, and *Zic5* were enriched in e-NMPs. The function of many of these genes will require further investigation because, for example, *Pou5f1* (*Oct3/4*) has recently been implicated in the maintenance of axial progenitors and trunk extension (Aires et al., 2016).

Alongside the core NMP signature, stage-specific gene expression was apparent. A set of genes including *Cdh1*, *Fst*, and *Grsf1* was present in e8.5 NMPs but downregulated by e9.5. The well-established spatial-temporal order of *Hox* gene expression was also evident. Accordingly, e8.5 NMPs expressed *3′Hox* genes, whereas e9.5 NMPs expressed more posterior 5′ *Hox* genes, notably *Hoxa9*, *Hoxc9*, *Hoxd9*, and *Hoxa10*. Apart from differences in *Hox* gene expression there was a clear difference in the expression of RA signaling pathway modulators. e8.5 NMPs expressed *Nr6a1*, a mediator of RA signaling in ESCs that is required for the exit from pluripotency (Akamatsu et al., 2009). By contrast, *Cyp26a1*, the RA-metabolizing enzyme, is upregulated in e9.5 NMPs. Together, the data provide a core gene expression signature of e-NMPs and identified changes that represent the embryonic age of NMPs.

During the differentiation of NMPs to PSM, cells transit through an MPC state in which *T/Bra*, *Nkx1.2*, *Msgn1*, and *Tbx6* are co-expressed. MPCs then downregulate T/*Bra* and *Nkx1.2*, adopting a PSM identity that is *Msgn1*$^+$/*Tbx6*$^+$ (Chalamalasetty et al., 2014). Cells with the molecular characteristics of MPC were evident from in vivo transcriptome data at both e8.5 and e9.5. In addition, cells with a PSM identity were observed from e8.5 embryos. This is consistent with the physical proximity of these cell types in the embryo. Moreover, a pseudotemporal ordering of cells, using the gene expression data, reconstructed the progression of NMPs to PSM. Hence, in addition to the embryonic time identified by comparison of e8.5 and e9.5 data, the developmental progression of NMPs to PSM was recovered from the in vivo analysis.

Conspicuously, D3 in vitro NMPs closely resembled e8.5 NMPs. In total, of the 68 genes comprising the e-NMP signature in vivo, 49 genes showed a similar behavior in vitro. Moreover, similar to in vivo, cells with an MPC identity were observed in vitro. The developmental trajectory reconstructed from the in vitro data mirrored that of the in vivo cells, indicating that succession of gene expression during NMP to PSM differentiation was similar in vitro as in vivo. Comparison of in vitro NMPs to e-NMPs also documented differences between the two populations. These included genes implicated in various aspects of metabolism and ribosome biogenesis. It seems likely that these differences relate to the culture conditions and the composition of the medium used in vitro. Irrespective of these differences, the overall correspondence in the core gene expression signature between in vitro NMPs and e-NMPs indicates that the in vitro population represents a valid model of in vivo NMPs.

Changes in the temporal axial identity of NMPs were recapitulated in vitro. At D3, in vitro NMPs were more similar to e8.5 NMPs than e9.5 NMPs. Removing *Tbx6* allowed the maintenance of in vitro NMPs for several days, and this resulted in the induction of markers characteristic of e9.5 NMPs. At D6, most *Tbx6*$^{-/-}$ cells expressed *Hoxc10*, and by D9 *Hox13* paralogs were activated (Figure 6G). It was notable that the proliferation of in vitro NMPs decreased over time and it proved impossible to maintain *Tbx6*$^{-/-}$ cells for longer than ~10 days. This appears to resemble the in vivo behavior of NMPs. The number of NMPs in an embryo peaks at e9.5-e10.5, then declines, and is exhausted by e13.5, coinciding with the increased expression of Hox13 proteins in the tailbud and the termination of axis elongation (Wymeersch et al., 2016; Denans et al., 2015; Young et al., 2009). These findings suggest that the elimination of NMPs is intrinsic and not dependent on external signals. This extends

the molecular and functional similarity between in vitro and in vivo NMPs and provides an experimentally tractable system in which to investigate the mechanisms controlling the exhaustion of axial progenitors and termination of axis elongation.

RA Signaling in the Establishment and Resolution of the NMP State

The reconstructed developmental trajectories highlighted the importance of RA in the formation and differentiation of NMPs. In embryos, the spatial distribution of RA signaling is controlled by the RA-synthesizing and degrading enzymes, *Raldh2* (*Aldh1a2*) and *Cyp26a1*, respectively. During much of axis elongation, the somites and anterior regions of the PSM express Raldh2 (Ribes et al., 2009; Sirbu and Duester, 2006). RA from these sources is implicated in neural induction and the transition of pre-neural cells into neural progenitors of the spinal cord (Diez del Corral et al., 2003). Consistent with this, the addition of RA to in vitro NMPs at D3 promotes neural differentiation.

Aldh1a2 is also expressed in the primitive streak, some node cells, and posterior mesoderm as early as e6.75, whereas response to RA signaling is reported from e7.5 (Sirbu et al., 2005; Ribes et al., 2009). Targeted disruption of the *Aldh1a2* gene in mouse embryos is associated with the shortening of the anterior-posterior axis (Niederreither et al., 1999; Cunningham et al., 2015; Duester, 2008; Niederreither and Dolle, 2008), whereas excess RA administration results in caudal axial truncations (Iulianella et al., 1999). Embryos lacking *RAR*γ are resistant to these RA-induced posterior defects, highlighting a unique role for this receptor (Lohnes et al., 1993; Janesick et al., 2014). These data argue for an early role of RA in axis elongation and the generation of NMPs, but definitive evidence had been missing due to the potential maternal contribution of RA in the *Aldh1a2*$^{-/-}$ mice. Analysis of the single-cell data showed that the *RAR*γ receptor is expressed in NMP cells in vivo and in vitro, whereas *Cyp26a1* is expressed in e9.5, but not e8.5 NMPs. Consistent with this *Cyp26a1* is upregulated in the tailbud at e9.0 (Sakai et al., 2001) and is proposed to maintain RA signaling at levels that allow the maintenance of NMPs. Strikingly, removal of RA signaling from D0 in vitro resulted in the loss of NMP identity, downregulation of Sox2 expression, and a significant upregulation of mesodermal specific genes. This included the induction of markers, such as *Eomes* and *Mixl1* (Figure S2A), characteristic of the early primitive streak that normally generates anterior mesoderm. Conversely, exposure of differentiating NMPs to high levels of RA blocked mesoderm induction. Instead the cells acquired a PNT identity characterized by the expression of *Sox2*, *Sox1*, and *Nkx1.2* (Figure 4D), consistent with recent studies identifying *Sox2* as an early responder to RA signaling in NMPs (Cunningham et al., 2016).

Taken together the data suggest that in vivo, a gradient of RA extends into the CLE and primitive streak to influence the induction and rostral-caudal positioning of distinct trunk progenitors. This gradient is composed of RA produced within the caudal epiblast together with RA diffusing from more anterior mesoderm sources, and is countered by Cyp26a1 expressed in posterior cells (Olivera-Martinez et al., 2012). The combination of the caudal flux of RA and local degradation would establish a rostral-to-caudal gradient of RA signaling. In the anterior region of the CLE low levels of RA signaling are required for the induction of NMP identity, whereas the absence of RA signaling in combination with high Wnt and Fgf signaling promotes T/Bra expression and an anterior mesodermal identity. Higher levels of RA anterior to the caudal epiblast blocks *T/Bra* induction resulting in the generation of PNT cells and the differentiation of neural progenitors (Olivera-Martinez et al., 2012). Collectively, therefore, the data argue that tightly controlled levels of RA signaling are essential for the induction and differentiation of NMPs. This explains the importance of RA in establishing the body axis and places renewed attention on understanding the integration of RA signaling with the other signaling pathways implicated in the development of cell types in the primitive streak.

Surprisingly, RA appears dispensable for the development of the zebrafish trunk (Alexa et al., 2009). This might reflect differences in the mechanism of axis elongation in different vertebrates (Steventon et al., 2016). In this context, understanding the function of FGF signaling may provide insight. FGF has been implicated in both neural and mesodermal induction (Stern, 2005; Naiche et al., 2011; Boulet and Capecchi, 2012), with both T/Bra and Sox2 identified as direct targets (Isaacs et al., 1994; Takemoto et al., 2006). Moreover, FGF signaling upregulates the expression of genes characteristic of NMPs, such as Nkx1.2, which in turn promote Fgf8 expression (Sasai et al., 2014). This suggests that FGF signaling plays a role in establishing the bipotentiality of NMPs, while RA and Wnt signaling promote resolution into neural and mesodermal fates, respectively.

A Regulatory Network Balances Neural and Mesodermal Differentiation

The ability to reconstruct developmental trajectories of both spinal cord and PSM allowed us to reverse engineer the NMP regulatory network and test the function of key factors. Mutations in the *Cdx* genes block the induction of posterior *Hox* genes and impair axial extension in embryos (Savory et al., 2009; Young et al., 2009; van de Ven et al., 2011; van Rooijen et al., 2012; Amin et al., 2016; Neijts et al., 2016). The truncation of the body axis can be partially rescued by exposure to Wnt or FGF signaling (Young et al., 2009; van Rooijen et al., 2012; van de Ven et al., 2011). Nevertheless, although the induction of *Wnt3a*, *Fgf8*, and *T/Bra* by Cdx proteins have been implicated in these phenotypes, the severity of morphological defects in mutant embryos has made it difficult to establish the relative importance of target genes and to rule out others. In vitro, the elimination of all three *Cdx* genes recapitulated in vivo observations: *Wnt3a*, *Fgf8*, and posterior *Hox* gene expression were lost. In addition, there was increased expression of *Aldh1a2* and a downregulation of *Cyp26a1*. Inhibition of RA signaling partially restored anterior mesoderm induction but did not restore NMPs. Thus, *Cdx* genes are necessary for the generation of functional NMPs. *Cdx* genes are required for the expression of trunk Hox genes, the expression of which can at least partially substitute for the activity of Cdx proteins (Young et al., 2009). Consequently, Hox proteins may function downstream or with *Cdx* genes in NMPs.

The TFs, *T/Bra*, *Tbx6*, and *Msgn1*, are also implicated in NMP development (Nowotschin et al., 2012; Chapman et al., 2003; Garriock et al., 2015). Separating the roles of *Msgn1* and *Tbx6* is complicated by the similarity in their expression and function.

In vivo, both genes are induced simultaneously and the loss of one results in reduced expression of the other (Nowotschin et al., 2012). Embryos lacking either *Msgn1* or *Tbx6* have defects in axis elongation, partial loss of mesodermal tissue, and enlarged tailbuds (Chapman et al., 2003; Chapman and Papaioannou, 1998; Yoon and Wold, 2000). *Tbx6* is important for the repression of *Sox2* during mesoderm specification (Li and Storey, 2011; Takemoto et al., 2011). Conversely, overexpression of *Msgn1* results in decreased neural induction (Chalamalasetty et al., 2014). In ESCs lacking either *Msgn1* or *Tbx6* genes, NMP identity could be induced with high efficiency, but exit from the NMP state and the induction of mesoderm differentiation was defective. Removal of *Msgn1* resulted in co-expression of T/Bra, Sox2, and Tbx6. This argues that *Tbx6* is not sufficient to repress *Sox2* in the absence of *Msgn1*. By contrast, in cells lacking *Tbx6*, expression of *Msgn1* was initially induced but not sustained. Instead, cells reverted to an NMP gene expression profile that was maintained for several days and multiple passages. In addition to the defects in mesoderm differentiation, *Aldh1a2* induction was also lost in *Tbx6* or *Msgn1* mutants. The reduction in RA signaling resulted in the maintenance of a progenitor identity suggesting that, once induced, RA signaling is dispensable for NMP maintenance. Collectively, the data suggest that in the presence of Wnt signaling, the loss of *Msgn1* blocks mesoderm differentiation at an MPC-like stage, whereas in the absence of Tbx6, cells do not progress beyond an NMP state.

We formulated a minimal gene regulatory model that accounts for the experimental observations and describes NMP formation and differentiation. In this model, Wnt signaling initiates a cascade by inducing *T/Bra* that represses *Sox2* and induces *Tbx6/Msgn1*. *T/Bra* and *Sox2* are then inhibited by *Tbx6/Msgn1* to establish a mesoderm state. *Tbx6/Msgn1* also activates RA production (by regulating *Aldh1a2*), which provides feedback to activate *Sox2*. The induction of *Sox2* inhibits *T/Bra* and *Tbx6/Msgn1* to establish neural identity. Hence, countervailing activities of Wnt and RA signaling, acting through mutually inhibiting *T/Bra* and *Sox2*, produces a switch that resolves into either mesoderm or neural differentiation. In the deterministic formulation of the model, the levels of Wnt and RA signaling dictates the outcome. The introduction of noise to the simulations, mimicking the stochastic nature of gene expression, produces a mixture of neural and mesodermal cells. The relative levels of RA and Wnt signaling control the proportion of the two cell types. In agreement with this, altering the levels of RA and Wnt signaling in differentiating NMPs in vitro determines the ratio of Sox2$^+$ neural and Tbx6$^+$ mesodermal cells generated.

From this point of view, the bipotential progenitor, corresponding to Sox2 and T/Bra co-expression, represents a metastable state in which two mutually inhibitory factors are activated in response to opposing signals. The expression of conflicting lineage-associated TFs has been referred to as multilineage priming and observed in other multipotent progenitors, including hematopoietic stem cells (Hu et al., 1997; Delassus et al., 1999; Enver and Greaves, 1998; Mansson et al., 2007) and ESCs (Hayashi et al., 2008; Laslett et al., 2007). For example, *Oct3/4* and *Sox2* maintain pluripotency of ESCs, but also drive the mutually exclusive differentiation to endoderm and neuroectoderm, respectively (Thomson et al., 2011). Thus the activities of *Oct3/4* and *Sox2* appear to parallel the function of *Sox2* and T/*Bra* in NMPs.

A salient feature of the NMP regulatory network is that RA production from Tbx6$^+$/Msgn1$^+$ mesoderm provides regulative feedback to NMPs. The differentiation of mesoderm increases RA production that, in turn, promotes *Sox2* expression in undifferentiated NMPs and thereby increases neural differentiation at the expense of mesoderm (Figure 3G). Conversely, a decrease in mesoderm reduces RA signaling, leading to a decrease in *Sox2* expression and a compensatory increase in mesoderm production. This reveals a design logic to the network architecture. Opposing signals establish a bipotent state that is resolved by cross-repressive transcriptional interactions between the induced TFs (Figure 3G). Regulative feedback from the progeny then ensures appropriate proportions of each tissue are generated. Thus, a feedback-modulated noisy bistable switch allows the balanced generation of two cell types from a bipotential progenitor and facilitates orderly axis elongation.

STAR★METHODS

Detailed methods are provided in the online version of this paper and include the following:

- KEY RESOURCES TABLE
- CONTACT FOR REAGENT AND RESOURCE SHARING
- EXPERIMENTAL MODEL AND SUBJECT DETAILS
- METHOD DETAILS
  - Dissection of NMP Cells from Mouse Embryos
  - Generation of Mouse Mutant ESC Lines Using the Crispr/Cas9 System
  - ESC Culture and Differentiation
  - Reverse Transcription – Quantitative PCR Analysis
  - Immunostaining - Antibodies
  - FACs Analysis
  - RNA-Sequencing and Data Pre-Processing
  - Dimension Reduction
  - Cell Population Clustering
  - Single-Cell State Graph
  - Pseudo-Temporal Ordering
  - Deterministic Model of Gene Regulation
  - Optimization Algorithm
  - Parameters, Initial State and External Conditions
  - Distance Function
  - Stochastic Model of Gene Regulation
- QUANTIFICATION AND STATISTICAL ANALYSIS
- DATA AND SOFTWARE AVAILABILITY

SUPPLEMENTAL INFORMATION

Supplemental Information includes four figures and five tables and can be found with this article online at http://dx.doi.org/10.1016/j.devcel.2017.04.002.

AUTHOR CONTRIBUTIONS

M.G. and J.B. conceived and designed the project and wrote the manuscript. M.G. performed and analyzed ESC differentiation experiments and generated the single-cell RNA-seq libraries. J.D. developed the single-cell data analysis methods and performed the computational modeling. D.S. designed and generated the Crispr ESC lines and performed ESC experiments. J.K.

developed the pipeline for single-cell RNA mapping. Y.H., F.J.W., and V.W. performed the dissections of NMP cells from mouse embryos.

ACKNOWLEDGMENTS

We thank Virginia E. Papaioannou for providing us with the Tbx6 mutant ESC line, Leena Bhaw-Rosun and Abdul Sesay for processing Illumina libraries, Robert Blassberg for assistance with FACs experiments, and Johanna Schmid and Neda Mousavy for technical assistance. This work was supported by the Francis Crick Institute, which receives its core funding from Cancer Research UK (FC001051), the UK Medical Research Council (FC001051), and the Wellcome Trust (FC001051); by the Wellcome Trust (grant reference WT098326MA); and by the BBSRC (grant reference BB/J015539/1). M.G. was also supported by the Max Delbrück Center for Molecular Medicine in the Helmholtz Association.

Received: September 21, 2016
Revised: February 20, 2017
Accepted: April 3, 2017
Published: April 27, 2017

REFERENCES

Aires, R., Jurberg, A.D., Leal, F., Novoa, A., Cohn, M.J., and Mallo, M. (2016). Oct4 is a key regulator of vertebrate trunk length diversity. Dev. Cell *38*, 262–274.

Akamatsu, W., DeVeale, B., Okano, H., Cooney, A.J., and van der Kooy, D. (2009). Suppression of Oct4 by germ cell nuclear factor restricts pluripotency and promotes neural stem cell development in the early neural lineage. J. Neurosci. *29*, 2113–2124.

Alexa, K., Choe, S.K., Hirsch, N., Etheridge, L., Laver, E., and Sagerstrom, C.G. (2009). Maternal and zygotic aldh1a2 activity is required for pancreas development in zebrafish. PLoS One *4*, e8261.

Amin, S., Neijts, R., Simmini, S., van Rooijen, C., Tan, S.C., Kester, L., van Oudenaarden, A., Creyghton, M.P., and Deschamps, J. (2016). Cdx and T brachyury co-activate growth signaling in the embryonic axial progenitor niche. Cell Rep. *17*, 3165–3177.

Anders, S., Pyl, P.T., and Huber, W. (2015). HTSeq – a Python framework to work with high-throughput sequencing data. Bioinformatics *31*, 166–169.

Ang, H.L., and Duester, G. (1997). Initiation of retinoid signaling in primitive streak mouse embryos: spatiotemporal expression patterns of receptors and metabolic enzymes for ligand synthesis. Dev. Dyn. *208*, 536–543.

Bouldin, C.M., Manning, A.J., Peng, Y.H., Farr, G.H., 3rd, Hung, K.L., Dong, A., and Kimelman, D. (2015). Wnt signaling and tbx16 form a bistable switch to commit bipotential progenitors to mesoderm. Development *142*, 2499–2507.

Boulet, A.M., and Capecchi, M.R. (2012). Signaling by FGF4 and FGF8 is required for axial elongation of the mouse embryo. Dev. Biol. *371*, 235–245.

Cambray, N., and Wilson, V. (2002). Axial progenitors with extensive potency are localised to the mouse chordoneural hinge. Development *129*, 4855–4866.

Cambray, N., and Wilson, V. (2007). Two distinct sources for a population of maturing axial progenitors. Development *134*, 2829–2840.

Camp, J.G., Badsha, F., Florio, M., Kanton, S., Gerber, T., Wilsch-Brauninger, M., Lewitus, E., Sykes, A., Hevers, W., Lancaster, M., et al. (2015). Human cerebral organoids recapitulate gene expression programs of fetal neocortex development. Proc. Natl. Acad. Sci. USA *112*, 15672–15677.

Csardi, G., and Nepusz, T. (2006). The igraph software package for complex network research. Inter. J. Complex Syst. 1695.

Chalamalasetty, R.B., Dunty, W.C., Jr., Biris, K.K., Ajima, R., Iacovino, M., Beisaw, A., Feigenbaum, L., Chapman, D.L., Yoon, J.K., Kyba, M., et al. (2011). The Wnt3a/beta-catenin target gene Mesogenin1 controls the segmentation clock by activating a Notch signalling program. Nat. Commun. *2*, 390.

Chalamalasetty, R.B., Garriock, R.J., Dunty, W.C., Jr., Kennedy, M.W., Jailwala, P., Si, H., and Yamaguchi, T.P. (2014). Mesogenin 1 is a master regulator of paraxial presomitic mesoderm differentiation. Development *141*, 4285–4297.

Chapman, D.L., Cooper-Morgan, A., Harrelson, Z., and Papaioannou, V.E. (2003). Critical role for Tbx6 in mesoderm specification in the mouse embryo. Mech. Dev. *120*, 837–847.

Chapman, D.L., and Papaioannou, V.E. (1998). Three neural tubes in mouse embryos with mutations in the T-box gene Tbx6. Nature *391*, 695–697.

Cunningham, T.J., Brade, T., Sandell, L.L., Lewandoski, M., Trainor, P.A., Colas, A., Mercola, M., and Duester, G. (2015). Retinoic acid activity in undifferentiated neural progenitors is sufficient to fulfill its role in restricting Fgf8 expression for somitogenesis. PLoS One *10*, e0137894.

Cunningham, T.J., Colas, A., and Duester, G. (2016). Early molecular events during retinoic acid induced differentiation of neuromesodermal progenitors. Biol. Open *5*, 1821–1833.

Davidson, E.H. (2010). Emerging properties of animal gene regulatory networks. Nature *468*, 911–920.

Delassus, S., Titley, I., and Enver, T. (1999). Functional and molecular analysis of hematopoietic progenitors derived from the aorta-gonad-mesonephros region of the mouse embryo. Blood *94*, 1495–1503.

Delfino-Machin, M., Lunn, J.S., Breitkreuz, D.N., Akai, J., and Storey, K.G. (2005). Specification and maintenance of the spinal cord stem zone. Development *132*, 4273–4283.

Denans, N., Iimura, T., and Pourquie, O. (2015). Hox genes control vertebrate body elongation by collinear Wnt repression. Elife *4*, 04379.

Deschamps, J., and van Nes, J. (2005). Developmental regulation of the Hox genes during axial morphogenesis in the mouse. Development *132*, 2931–2942.

Diez del Corral, R., Olivera-Martinez, I., Goriely, A., Gale, E., Maden, M., and Storey, K. (2003). Opposing FGF and retinoid pathways control ventral neural pattern, neuronal differentiation, and segmentation during body axis extension. Neuron *40*, 65–79.

Duester, G. (2008). Retinoic acid synthesis and signaling during early organogenesis. Cell *134*, 921–931.

Evans, M.J., and Kaufman, M.H. (1981). Establishment in culture of pluripotential cells from mouse embryos. Nature *292*, 154–156.

Enver, T., and Greaves, M. (1998). Loops, lineage, and leukemia. Cell *94*, 9–12.

Fisher, W. (1958). On grouping for maximum homogeneity. J. Am. Stat. Assoc. *53*, 789–798.

Garriock, R.J., Chalamalasetty, R.B., Kennedy, M.W., Canizales, L.C., Lewandoski, M., and Yamaguchi, T.P. (2015). Lineage tracing of neuromesodermal progenitors reveals novel Wnt-dependent roles in trunk progenitor cell maintenance and differentiation. Development *142*, 1628–1638.

Gouti, M., Tsakiridis, A., Wymeersch, F.J., Huang, Y., Kleinjung, J., Wilson, V., and Briscoe, J. (2014). In vitro generation of neuromesodermal progenitors reveals distinct roles for wnt signalling in the specification of spinal cord and paraxial mesoderm identity. PLoS Biol. *12*, e1001937.

Gouti, M., Metzis, V., and Briscoe, J. (2015). The route to spinal cord cell types: a tale of signals and switches. Trends Genet. *31*, 282–289.

Hayashi, K., Lopes, S.M., Tang, F., and Surani, M.A. (2008). Dynamic equilibrium and heterogeneity of mouse pluripotent stem cells with distinct functional and epigenetic states. Cell Stem Cell *3*, 391–401.

Henrique, D., Abranches, E., Verrier, L., and Storey, K.G. (2015). Neuromesodermal progenitors and the making of the spinal cord. Development *142*, 2864–2875.

Herrmann, B.G., Labeit, S., Poustka, A., King, T.R., and Lehrach, H. (1990). Cloning of the T gene required in mesoderm formation in the mouse. Nature *343*, 617–622.

Hu, M., Krause, D., Greaves, M., Sharkis, S., Dexter, M., Heyworth, C., and Enver, T. (1997). Multilineage gene expression precedes commitment in the hemopoietic system. Genes Dev. *11*, 774–785.

Isaacs, H.V., Pownall, M.E., and Slack, J.M. (1994). eFGF regulates Xbra expression during Xenopus gastrulation. EMBO J. *13*, 4469–4481.

Iulianella, A., Beckett, B., Petkovich, M., and Lohnes, D. (1999). A molecular basis for retinoic acid-induced axial truncation. Dev. Biol. *205*, 33–48.

Janesick, A., Nguyen, T.T., Aisaki, K., Igarashi, K., Kitajima, S., Chandraratna, R.A., Kanno, J., and Blumberg, B. (2014). Active repression by RARgamma signaling is required for vertebrate axial elongation. Development *141*, 2260–2270.

Kessel, M. (1992). Respecification of vertebral identities by retinoic acid. Development *115*, 487–501.

Kim, D., Pertea, G., Trapnell, C., Pimentel, H., Kelley, R., and Salzberg, S.L. (2013). TopHat2: accurate alignment of transcriptomes in the presence of insertions, deletions and gene fusions. Genome Biol. *14*, R36.

Kimelman, D. (2016). Tales of tails (and trunks): forming the posterior body in vertebrate embryos. Curr. Top Dev. Biol. *116*, 517–536.

Kondoh, H., and Takemoto, T. (2012). Axial stem cells deriving both posterior neural and mesodermal tissues during gastrulation. Curr. Opin. Genet. Dev. *22*, 374–380.

Langmead, B., and Salzberg, S.L. (2012). Fast gapped-read alignment with Bowtie 2. Nat. Methods *9*, 357–359.

Laslett, A.L., Grimmond, S., Gardiner, B., Stamp, L., Lin, A., Hawes, S.M., Wormald, S., Nikolic-Paterson, D., Haylock, D., and Pera, M.F. (2007). Transcriptional analysis of early lineage commitment in human embryonic stem cells. BMC Dev. Biol. *7*, 12.

Li, R.A., and Storey, K.G. (2011). An emerging molecular mechanism for the neural vs mesodermal cell fate decision. Cell Res. *21*, 708–710.

Lickert, H., Cox, B., Wehrle, C., Taketo, M.M., Kemler, R., and Rossant, J. (2005). Dissecting Wnt/beta-catenin signaling during gastrulation using RNA interference in mouse embryos. Development *132*, 2599–2609.

Lippmann, E.S., Williams, C.E., Ruhl, D.A., Estevez-Silva, M.C., Chapman, E.R., Coon, J.J., and Ashton, R.S. (2015). Deterministic HOX patterning in human pluripotent stem cell-derived neuroectoderm. Stem Cell Rep. *4*, 632–644.

Lohnes, D., Kastner, P., Dierich, A., Mark, M., LeMeur, M., and Chambon, P. (1993). Function of retinoic acid receptor gamma in the mouse. Cell *73*, 643–658.

Magin, T.M., McWhir, J., and Melton, D.W. (1992). A new mouse embryonic stem cell line with good germ line contribution and gene targeting frequency. Nucleic Acids Res. *20*, 3795–3796.

Mansson, R., Hultquist, A., Luc, S., Yang, L., Anderson, K., Kharazi, S., Al-Hashmi, S., Liuba, K., Thoren, L., Adolfsson, J., et al. (2007). Molecular evidence for hierarchical transcriptional lineage priming in fetal and adult stem cells and multipotent progenitors. Immunity *26*, 407–419.

Martin, B.L., and Kimelman, D. (2008). Regulation of canonical Wnt signaling by Brachyury is essential for posterior mesoderm formation. Dev. Cell *15*, 121–133.

Martin, B.L., and Kimelman, D. (2010). Brachyury establishes the embryonic mesodermal progenitor niche. Genes Dev. *24*, 2778–2783.

Martin, B.L., and Kimelman, D. (2012). Canonical Wnt signaling dynamically controls multiple stem cell fate decisions during vertebrate body formation. Dev. Cell *22*, 223–232.

Martins, E., and Pascoal, M. (2003). A new implementation of Yen's ranking loopless paths algorithm. Quarterly Journal of the Belgian French and Italian Operations Research Societies *1*, 121–133.

Mazzoni, E.O., Mahony, S., Peljto, M., Patel, T., Thornton, S.R., McCuine, S., Reeder, C., Boyer, L.A., Young, R.A., Gifford, D.K., et al. (2013). Saltatory remodeling of Hox chromatin in response to rostrocaudal patterning signals. Nat. Neurosci. *16*, 1191–1198.

Mc Mahon, S.S., Sim, A., Filippi, S., Johnson, R., Liepe, J., Smith, D., and Stumpf, M.P. (2014). Information theory and signal transduction systems: from molecular information processing to network inference. Semin. Cell Dev. Biol. *35*, 98–108.

Naiche, L.A., Holder, N., and Lewandoski, M. (2011). FGF4 and FGF8 comprise the wavefront activity that controls somitogenesis. Proc. Natl. Acad. Sci. USA *108*, 4018–4023.

Neijts, R., Simmini, S., Giuliani, F., van Rooijen, C., and Deschamps, J. (2014). Region-specific regulation of posterior axial elongation during vertebrate embryogenesis. Dev. Dyn. *243*, 88–98.

Neijts, R., Amin, S., van Rooijen, C., and Deschamps, J. (2016). Cdx is crucial for the timing mechanism driving colinear Hox activation and defines a trunk segment in the Hox cluster topology. Dev. Biol. *422*, 146–154.

Niederreither, K., and Dolle, P. (2008). Retinoic acid in development: towards an integrated view. Nat. Rev. Genet. *9*, 541–553.

Niederreither, K., McCaffery, P., Drager, U.C., Chambon, P., and Dolle, P. (1997). Restricted expression and retinoic acid-induced downregulation of the retinaldehyde dehydrogenase type 2 (RALDH-2) gene during mouse development. Mech. Dev. *62*, 67–78.

Niederreither, K., Subbarayan, V., Dolle, P., and Chambon, P. (1999). Embryonic retinoic acid synthesis is essential for early mouse post-implantation development. Nat. Genet. *21*, 444–448.

Nordstrom, U., Maier, E., Jessell, T.M., and Edlund, T. (2006). An early role for WNT signaling in specifying neural patterns of Cdx and Hox gene expression and motor neuron subtype identity. PLoS Biol. *4*, e252.

Nowotschin, S., Ferrer-Vaquer, A., Concepcion, D., Papaioannou, V.E., and Hadjantonakis, A.K. (2012). Interaction of Wnt3a, Msgn1 and Tbx6 in neural versus paraxial mesoderm lineage commitment and paraxial mesoderm differentiation in the mouse embryo. Dev. Biol. *367*, 1–14.

Olivera-Martinez, I., Harada, H., Halley, P.A., and Storey, K.G. (2012). Loss of FGF-dependent mesoderm identity and rise of endogenous retinoid signalling determine cessation of body axis elongation. Plos Biol. *10*, e1001415.

Oulad-Abdelghani, M., Chazaud, C., Bouillet, P., Sapin, V., Chambon, P., and Dolle, P. (1997). Meis2, a novel mouse Pbx-related homeobox gene induced by retinoic acid during differentiation of P19 embryonal carcinoma cells. Dev. Dyn. *210*, 173–183.

Patel, A.P., Tirosh, I., Trombetta, J.J., Shalek, A.K., Gillespie, S.M., Wakimoto, H., Cahill, D.P., Nahed, B.V., Curry, W.T., Martuza, R.L., et al. (2014). Single-cell RNA-seq highlights intratumoral heterogeneity in primary glioblastoma. Science *344*, 1396–1401.

Parks, D.R., Roederer, M., and Moore, W.A. (2006). A new "Logicle" display method avoids deceptive effects of logarithmic scaling for low signals and compensated data. Cytometry A *69*, 541–551.

Perez-Carrasco, R., Guerrero, P., Briscoe, J., and Page, K.M. (2016). Intrinsic noise profoundly alters the dynamics and steady state of morphogen-controlled bistable genetic switches. PLoS Comput. Biol. *12*, e1005154.

Ran, F.A., Hsu, P.D., Wright, J., Agarwala, V., Scott, D.A., and Zhang, F. (2013). Genome engineering using the CRISPR-Cas9 system. Nat. Protoc. *8*, 2281–2308.

Ribes, V., Le Roux, I., Rhinn, M., Schuhbaur, B., and Dolle, P. (2009). Early mouse caudal development relies on crosstalk between retinoic acid, Shh and Fgf signalling pathways. Development *136*, 665–676.

Sakai, Y., Meno, C., Fujii, H., Nishino, J., Shiratori, H., Saijoh, Y., Rossant, J., and Hamada, H. (2001). The retinoic acid-inactivating enzyme CYP26 is essential for establishing an uneven distribution of retinoic acid along the anterio-posterior axis within the mouse embryo. Genes Dev. *15*, 213–225.

Sasai, N., Kutejova, E., and Briscoe, J. (2014). Integration of signals along orthogonal axes of the vertebrate neural tube controls progenitor competence and increases cell diversity. Plos Biol. *12*, e1001907.

Satija, R., Farrell, J.A., Gennert, D., Schier, A.F., and Regev, A. (2015). Spatial reconstruction of single-cell gene expression data. Nat. Biotechnol. *33*, 495–502.

Savory, J.G., Bouchard, N., Pierre, V., Rijli, F.M., De Repentigny, Y., Kothary, R., and Lohnes, D. (2009). Cdx2 regulation of posterior development through non-Hox targets. Development *136*, 4099–4110.

Schindelin, J., Arganda-Carreras, I., Frise, E., Kaynig, V., Longair, M., Pietzsch, T., Preibisch, S., Rueden, C., Saalfeld, S., Schmid, B., et al. (2012). Fiji: an open-source platform for biological-image analysis. Nat. Methods *9*, 676–682.

Shea, M.A., and Ackers, G.K. (1985). The OR control system of bacteriophage lambda. A physical-chemical model for gene regulation. J. Mol. Biol. *181*, 211–230.

Sherman, M.S., and Cohen, B.A. (2012). Thermodynamic state ensemble models of *cis*-regulation. PLoS Comput. Biol. *8*, e1002407.

Shum, A.S., Poon, L.L., Tang, W.W., Koide, T., Chan, B.W., Leung, Y.C., Shiroishi, T., and Copp, A.J. (1999). Retinoic acid induces down-regulation of Wnt-3a, apoptosis and diversion of tail bud cells to a neural fate in the mouse embryo. Mech. Dev. *84*, 17–30.

Sirbu, I.O., and Duester, G. (2006). Retinoic-acid signalling in node ectoderm and posterior neural plate directs left-right patterning of somitic mesoderm. Nat. Cell Biol *8*, 271–277.

Sirbu, I.O., Gresh, L., Barra, J., and Duester, G. (2005). Shifting boundaries of retinoic acid activity control hindbrain segmental gene expression. Development *132*, 2611–2622.

Stern, C.D. (2005). Neural induction: old problem, new findings, yet more questions. Development *132*, 2007–2021.

Stern, C.D., Charite, J., Deschamps, J., Duboule, D., Durston, A.J., Kmita, M., Nicolas, J.F., Palmeirim, I., Smith, J.C., and Wolpert, L. (2006). Head-tail patterning of the vertebrate embryo: one, two or many unresolved problems? Int. J. Dev. Biol. *50*, 3–15.

Steventon, B., Duarte, F., Lagadec, R., Mazan, S., Nicolas, J.F., and Hirsinger, E. (2016). Species-specific contribution of volumetric growth and tissue convergence to posterior body elongation in vertebrates. Development *143*, 1732–1741.

Storn, R., and Price, K. (1997). Differential evolution – a simple and efficient heuristic for global optimization over continuous spaces. J. Glob. Optimization *11*, 341–359.

Takada, S., Stark, K.L., Shea, M.J., Vassileva, G., McMahon, J.A., and McMahon, A.P. (1994). Wnt-3a regulates somite and tailbud formation in the mouse embryo. Genes Dev. *8*, 174–189.

Takemoto, T., Uchikawa, M., Kamachi, Y., and Kondoh, H. (2006). Convergence of Wnt and FGF signals in the genesis of posterior neural plate through activation of the Sox2 enhancer N-1. Development *133*, 297–306.

Takemoto, T., Uchikawa, M., Yoshida, M., Bell, D.M., Lovell-Badge, R., Papaioannou, V.E., and Kondoh, H. (2011). Tbx6-dependent Sox2 regulation determines neural or mesodermal fate in axial stem cells. Nature *470*, 394–398.

Tenin, G., Wright, D., Ferjentsik, Z., Bone, R., McGrew, M.J., and Maroto, M. (2010). The chick somitogenesis oscillator is arrested before all paraxial mesoderm is segmented into somites. BMC Dev. Biol. *10*, 24.

Thomson, M., Liu, S.J., Zou, L.N., Smith, Z., Meissner, A., and Ramanathan, S. (2011). Pluripotency factors in embryonic stem cells regulate differentiation into germ layers. Cell *145*, 875–889.

Trapnell, C., Cacchiarelli, D., Grimsby, J., Pokharel, P., Li, S., Morse, M., Lennon, N.J., Livak, K.J., Mikkelsen, T.S., and Rinn, J.L. (2014). The dynamics and regulators of cell fate decisions are revealed by pseudotemporal ordering of single cells. Nat. Biotechnol. *32*, 381–386.

Tsakiridis, A., and Wilson, V. (2015). Assessing the bipotency of in vitro-derived neuromesodermal progenitors. F1000Res *4*, 100.

Tsakiridis, A., Huang, Y., Blin, G., Skylaki, S., Wymeersch, F., Osorno, R., Economou, C., Karagianni, E., Zhao, S., Lowell, S., et al. (2014). Distinct Wnt-driven primitive streak-like populations reflect in vivo lineage precursors. Development *141*, 1209–1221.

Tsakiridis, A., Huang, Y., Blin, G., Skylaki, S., Wymeersch, F., Osorno, R., Economou, C., Karagianni, E., Zhao, S., Lowell, S., et al. (2015). Distinct Wnt-driven primitive streak-like populations reflect in vivo lineage precursors. Development *142*, 809.

Turner, D.A., Hayward, P.C., Baillie-Johnson, P., Rue, P., Broome, R., Faunes, F., and Martinez Arias, A. (2014). Wnt/beta-catenin and FGF signalling direct the specification and maintenance of a neuromesodermal axial progenitor in ensembles of mouse embryonic stem cells. Development *141*, 4243–4253.

Tzouanacou, E., Wegener, A., Wymeersch, F.J., Wilson, V., and Nicolas, J.F. (2009). Redefining the progression of lineage segregations during mammalian embryogenesis by clonal analysis. Dev. Cell *17*, 365–376.

van de Ven, C., Bialecka, M., Neijts, R., Young, T., Rowland, J.E., Stringer, E.J., Van Rooijen, C., Meijlink, F., Novoa, A., Freund, J.N., et al. (2011). Concerted involvement of Cdx/Hox genes and Wnt signaling in morphogenesis of the caudal neural tube and cloacal derivatives from the posterior growth zone. Development *138*, 3451–3462.

van den Akker, E., Forlani, S., Chawengsaksophak, K., de Graaff, W., Beck, F., Meyer, B.I., and Deschamps, J. (2002). Cdx1 and Cdx2 have overlapping functions in anteroposterior patterning and posterior axis elongation. Development *129*, 2181–2193.

van Rooijen, C., Simmini, S., Bialecka, M., Neijts, R., van de Ven, C., Beck, F., and Deschamps, J. (2012). Evolutionarily conserved requirement of Cdx for post-occipital tissue emergence. Development *139*, 2576–2583.

Vidigal, J.A., Morkel, M., Wittler, L., Brouwer-Lehmitz, A., Grote, P., Macura, K., and Herrmann, B.G. (2010). An inducible RNA interference system for the functional dissection of mouse embryogenesis. Nucleic Acids Res. *38*, e122.

Wilson, V., Olivera-Martinez, I., and Storey, K.G. (2009). Stem cells, signals and vertebrate body axis extension. Development *136*, 1591–1604.

Wymeersch, F.J., Huang, Y., Blin, G., Cambray, N., Wilkie, R., Wong, F.C., and Wilson, V. (2016). Position-dependent plasticity of distinct progenitor types in the primitive streak. Elife *5*, e10042.

Yabe, T., and Takada, S. (2012). Mesogenin causes embryonic mesoderm progenitors to differentiate during development of zebrafish tail somites. Dev. Biol. *370*, 213–222.

Yamaguchi, T.P., Takada, S., Yoshikawa, Y., Wu, N., and McMahon, A.P. (1999). T (Brachyury) is a direct target of Wnt3a during paraxial mesoderm specification. Genes Dev. *13*, 3185–3190.

Ying, Q.L., Wray, J., Nichols, J., Batlle-Morera, L., Doble, B., Woodgett, J., Cohen, P., and Smith, A. (2008). The ground state of embryonic stem cell self-renewal. Nature *453*, 519–523.

Yoon, J.K., and Wold, B. (2000). The bHLH regulator pMesogenin1 is required for maturation and segmentation of paraxial mesoderm. Genes Dev. *14*, 3204–3214.

Yoon, J.K., Moon, R.T., and Wold, B. (2000). The bHLH class protein pMesogenin1 can specify paraxial mesoderm phenotypes. Dev. Biol. *222*, 376–391.

Yoshikawa, Y., Fujimori, T., McMahon, A.P., and Takada, S. (1997). Evidence that absence of Wnt-3a signaling promotes neuralization instead of paraxial mesoderm development in the mouse. Dev. Biol. *183*, 234–242.

Young, T., Rowland, J.E., van de Ven, C., Bialecka, M., Novoa, A., Carapuco, M., van Nes, J., de Graaff, W., Duluc, I., Freund, J.N., et al. (2009). Cdx and Hox genes differentially regulate posterior axial growth in mammalian embryos. Dev. Cell *17*, 516–526.

Zemel, R.S., and Carreira-Perpinan, M.A. (2005). Proximity graphs for clustering and manifold learning. Adv. Neural Inf. Process. Syst. *17*, 225–232.

CellPress

STAR★METHODS

KEY RESOURCES TABLE

REAGENT or RESOURCE	SOURCE	IDENTIFIER
Antibodies		
Goat anti-T/Bra	R&D	Cat# AF2085; RRID: AB_2200235
Goat anti-T/Bra	R&D	Cat# NL001; RRID: AB_663766
Goat anti-Tbx6	R&D	Cat# AF4744; RRID: AB_2200834
Rabbit anti-Sox2	R&D	Cat# AB5603; RRID: AB_304980
Rabbit anti-Hoxc10	Abcam	Cat# AB153904;
Mouse anti-Tuj1	Covance	Cat# MMS-435P; RRID: AB_2313773
Chemicals, Peptides, and Recombinant Proteins		
bFGF	R&D	3139-FB
CHIR99021	Axon	1386
BMS493	Tocris	3509
All-trans-RA	Sigma	2625
Critical Commercial Assays		
C1 Single-Cell Reagent Kit for mRNA Seq	Fluidigm	100-6201
C1 Single-Cell Auto Prep Array for mRNA-seq	Fluidigm	100-6041
Nextera XT index kit	Illumina	FC-131-1002
Nextera XT DNA Sample Preparation Kit	Illumina	FC-131-1096
SMARTer Ultra Low RNA Kit for the Fluidigm C1 System	Takara	634833
Alexa Fluor Antibody labeling kit	Molecular Probes	A20181
Deposited Data		
Single-cell RNA-seq data	This study	ArrayExpress: E-MTAB-5208
Experimental Models: Cell Lines		
HM1 mESCs	Thermo Scientific	N/A
Aldh1a2$^{-/-}$ mESCs	This study	N/A
Msgn1$^{-/-}$ mESCs	This study	N/A
Cdx$^{1,2,4,-/-}$ mESCs	This study	N/A
Tbx6$^{-/-}$ mESCs	Papaioannou VE Lab	Mech Dev. 2003 Jul;120(7):837-47
Experimental Models: Organisms/Strains		
Mouse: MF1	Biomedical Research Facility at the University of Edinburgh	N/A
Oligonucleotides		
Crispr Guide Cdx1: GGGGCCCGAAGGCCAGC	This study	N/A
Crispr Guide Cdx2: GCGCGTGGTATTCGGCG	This study	N/A
Crispr Guide Cdx4: GGATCCGAAAACTGGGG	This study	N/A
Crispr Guide Msgn1: GGCTGTAGACAGGCGGC	This study	N/A

(*Continued on next page*)

Continued		
REAGENT or RESOURCE	SOURCE	IDENTIFIER
Crispr Guide Aldh1a2: GAGTCATCAAAACCCTG	This study	N/A
qRT-PCR Primers	This study	Table S5
Recombinant DNA		
Plasmid pSpCas9/(BB)-2APuro	Addgene	48139
Software and Algorithms		
Fiji	Schindelin et al., 2012	https://imagej.net/Fiji
GraphPad Prism 6	GraphPad	https://graphpad.com/scientific-software/prism/
Bowtie2 v2.1.0	Langmead and Salzberg, 2012	http://bowtie-bio.sourceforge.net/bowtie2/index.shtml
Tophat v2.0.9	Kim et al., 2013	http://ccb.jhu.edu/software/tophat/index.shtml
HTSeq v0.5.4p3	Anders et al., 2015	http://www-huber.embl.de/HTSeq/doc/count.html
R v3.2.5	The R Foundation	https://www.r-project.org/
Flowcore	N/A	https://www.bioconductor.org/packages/release/bioc/html/flowCore.html
monocle v1.4.0 (R package)	Trapnell et al., 2014	https://bioconductor.org/packages/release/bioc/html/monocle.html
igraph v1.0.1 (R package)	Csardi and Nepusz, 2006	https://cran.r-project.org/web/packages/igraph/index.html
Rtsne v0.11 (R package)	N/A	https://cran.r-project.org/web/packages/Rtsne/index.html
Python v2.7.12	Python Software Foundation	http://www.python.org
Scipy (Python package)	N/A	http://www.scipy.org/

CONTACT FOR REAGENT AND RESOURCE SHARING

Further information and requests for reagents may be directed to, and will be fulfilled by, the Lead Contact, James Briscoe (James.Briscoe@crick.ac.uk).

EXPERIMENTAL MODEL AND SUBJECT DETAILS

Animal experiments were performed under the UK Home Office project licenses PD4515DD17 and PPL60/4435, approved by the Animal Welfare and Ethical Review Panel of the Francis Crick Institute and MRC Centre for Regenerative Medicine and within the conditions of the Animals (Scientific Procedures) Act 1986.

METHOD DETAILS

Dissection of NMP Cells from Mouse Embryos

Wildtype, outbred MF1 mice were used for timed matings. The noon on the day of finding a vaginal plug was designated e0.5 and embryos were collected at 8.5d.p.c and 9.5d.p.c. The caudal lateral epiblast region (L1-L2) was manually micro-dissected from e8.5 and e9.5 mouse embryos (Figure 1A; Wymeersch et al., 2016). The dissections removed the midline and the majority of underlying paraxial mesoderm cells using hand-pulled solid glass needles. The end product was mostly CLE with some underlying paraxial mesoderm cells. From each stage, the dissected regions were pooled separately and dissociated into single cells using a combination of enzyme (0.05% trypsin/EDTA) and mechanical (mouth pipetting) dissociation.

Generation of Mouse Mutant ESC Lines Using the Crispr/Cas9 System

We generated 3 different mutant mESC lines using the Crispr/Cas9 method (*Aldh1a2*$^{-/-}$, *Msgn1*$^{-/-}$ and *Cdx*$^{1,2,4-/-}$ line). Different guide RNAs were designed to target each gene using the CRISPR/Cas9 design tool (crispr.mit.edu.au) (STAR Methods). Each guide was cloned into the pSpCas9/(BB)-2APuro vector (Addgene 48139; Ran et al., 2013).

The wild type ESC line, HM1 (Magin et al., 1992) was maintained in ES cell medium (Evans and Kaufman, 1981) with 1000U/ml LIF (Chemicon) on mitotically inactive primary mouse embryo fibroblasts. For the electroporation, ESCs were removed from feeders by dissociation using 0.05% trypsin and then plated onto gelatinised tissue culture plates for two short successive periods (20-30mins) to remove feeder cells. Electroporation was performed using $4x10^6$ ESCs and 4 ug of CRISPR-Cas9 plasmid DNA (specific guide) using Amaxa cell line nucleofector kit (Lonza) according to the manufacturer's instructions (program A23). After electroporation cells were plated on Cell-Bind 10cm dishes with 2i medium/LIF (Ying et al., 2008). Puromycin selection was started 24 hours post-transfection by the addition of 1.5ug/ml puromycin for 48hours. Cells were allowed to grow for 5-7 days in 2i medium (Ying et al., 2008) and

the medium was changed every other day. Resulting colonies were picked manually and expanded in 2i medium plus LIF for genotyping. Clones were screened using specific PCR primers and then sequenced to confirm the presence of indels that introduce frameshift mutations and as a result early stop codons. The cell lines were tested for the expression of pluripotent markers.

ESC Culture and Differentiation

The mouse ESC lines, HM1 (WT) (Magin et al., 1992), Aldh1a2$^{-/-}$, Msgn1$^{-/-}$, Cdx$^{1,2,4-/-}$and Tbx6$^{-/-}$ (Chapman et al., 2003) were maintained in ESC medium (Evans and Kaufman, 1981) with 1000U/ml LIF (Chemicon) on mitotically inactive primary mouse embryo fibroblasts. To remove feeders, ESCs were dissociated using 0.05% trypsin and plated onto gelatinised tissue culture plates for two short successive periods (20-30mins). To induce differentiation, the cells were plated on Cell-Bind surface dishes (Corning) pre-coated with 0.1% gelatin (Sigma) at a density of 5-8x10^3 cells cm^{-2} in 'N2B27' medium (NB). N2B27 is a 1:1 medium of Advanced Dulbecco's Modified Eagle Medium F12 supplemented with 1 x N2 (Gibco), and Neurobasal medium (Gibco) supplemented with 1 x B27 (Gibco) or B27 minus Vitamin A (Gibco), 2mM L-glutamine (Gibco), 40μg/ml BSA fraction V (Sigma), 0.1mM 2-mercaptoethanol. Cells were grown in N2B27 supplemented with 10ng/ml bFgf (R&D) for 2 days. To induce neuromesodermal differentiation the cells were treated with 5uM CHIR99021 (Axon Medchem) and 10ng/ml bFgf for 24hours (D2-D3). After D3 the cells were transferred in N2B27 medium for neural differentiation or in N2B27 with 5uM CHIR99021 for mesodermal differentiation. For the maintenance of NMP identity, cells were passaged at a ratio 1:4 every 3 days on 35mm Cell-Bind dishes coated with 0.1% gelatin in N2B27 medium supplemented with 5uM CHIR99021 and 10ng/ml bFgf. The cells were dissociated into small cellular clumps mechanically using a plastic pipette tip. The medium was changed every other day.

Reverse Transcription – Quantitative PCR Analysis

Total RNA was isolated from cells using the RNeasy kit (Qiagen) according to the manufacturers instructions and digested with DNase I (Qiagen) to remove genomic DNA. First strand cDNA synthesis was performed with Superscript III system (Invitrogen) using random primers and amplified using Platinum SYBR-Green (Invitrogen). For QPCR the Applied Biosystems 7900HT Fast Real time PCR or the Light Cycler 480 SYBR Green I Master Mix (Roche) systems were used. PCR primers were designed using Primer3 software (See Table S5). All experiments were performed in biological duplicates or triplicates for each time point analysed. Expression values were normalized against the β-actin and standard deviations calculated and plotted using Prism 6 software (GraphPad).

Immunostaining - Antibodies

For immunostaining cells were fixed for 15mins at 4°C in 4% paraformaldehyde in phosphate buffer saline (PBS), then washed in PBST (PBS with 0.1% Triton-X100). The cells were blocked for 30mins at RT using blocking buffer A (1% BSA/0.1% Triton-X100 in PBS). Cells were incubated with primary antibodies o/n at 4°C and with secondary antibodies at room temperature for 2 hours. For sequential immunostaining with two goat antibodies after the end of the 1st immunostaining, cells were incubated with blocking buffer B (1% goat serum, 1% BSA, 0.1% Triton-X100 in PBS) for 1h at RT. The second goat antibody was directly conjugated with a fluorophore (Bra–NL557). Cells were incubated with the goat-conjugated antibody for 2h at RT in blocking buffer B. After washing with blocking buffer A the cells were mounted with DAPI containing Prolong Antifade (Molecular Probes). The following primary antibodies were used: goat anti-Brachyury (1:500) (R&D), goat anti- BraNL557 (1:250) (R&D), goat anti-Tbx6 (1:200) (R&D), rabbit anti-Sox2 (1:500) (Millipore), mouse anti-Tuj1 (1:1000) (Covance), mouse anti-Hoxc10 (1:200) (Abcam). The fluorescent images were captured using an inverted Leica SP5 confocal microscope.

FACs Analysis

Cells were dissociated into a single cell suspension using accutase (Gibco) for 5mins at 37°C. After washing with PBS, cells were fixed with 4% ice cold PFA on ice for 15mins. After washing with PBST (0.1% Triton-X100) the cells were incubated for 30min at RT in 100ul-200ul blocking solution (3%BSA / 0.2% Triton-X100 in PBS). The cells were treated with fluorescently conjugated primary antibodies for 1 hour at RT. Specifically, we used Sox2 conjugated to BD Horizon V450 (BD biosciences), T/Bra conjugated to NL557 (R&D) and Tbx6 (R&D) conjugated to Alexa Fluor 488. Conjugation of Tbx6 was performed using the Alexa Fluor 488 labelling kit (Thermofisher scientific) according to the manufacturer's instructions. Conjugated antibodies were diluted 1:100 in blocking solution and incubated for 1hr at RT on a shaker. Cells were washed with PBST for 3 times. Finally, cells were filtered with 200ul PBS in a BD Facs tube. Data acquisition was performed on a BD FACSVerse flow cytometer.

To analyse gene expression levels from the FACS data, we first removed debris cells on the basis of the forward scatter-area (FSC-A) and side scatter-area signals (SSC-A). Then we removed doublets using the FSC-A and forward scatter-height (FSC-H) signals. To improve the separation of cells with minimal fluorescence, we trained and applied a "logicle" transform (Parks et al., 2006) on channels measuring Bra, Sox2 and Tbx6 levels. To do this, we used the *estimateLogicle* function from the *flowCore* R package.

To identify the populations contained in samples of our in vitro differentiation protocol, we performed a k-means clustering ($k = 4$) in expression space (Bra, Sox2, Tbx6) of samples from each of the D3 and D4 samples. Because the number of D4 cells were greater than D3 (128177 cells versus 16631 cells), the clustering was performed after randomly downsampling the D4 samples to match the D3 sample size. The center coordinates of the 4 resulting clusters clearly demarcate their identity: (Bra^{HIGH}/Sox2^{LOW}) for NMPs, (Bra^{LOW}/Sox2^{LOW}) for mesodermal cells, (Bra^{LOW}/Sox2^{HIGH}) for neural progenitors and the fourth (Bra^{LOW}/Sox2^{LOW}) cluster center was "unassigned" population. Cells from each individual sample were then associated to the closest cluster center.

RNA-Sequencing and Data Pre-Processing

The single cells were captured using the C1 Single-Cell Auto Prep Integrated Fluidic Circuit (IFC) (10-17um). RNA and cDNA was prepared from single cells using the SMARTer ultra low RNA kit (Clontech). The RNA-Seq libraries were prepared using the Nextera XT DNA library prep kit (Illumina) according to manufacturers instructions described in the protocol (PN 100-7168, http://www.fluidigm.com/). Library size, purity and concentration were determined using Agilent Technologies 2100 Bioanalyzer. The libraries from 70-80 individual cells were pooled and sequenced using the Illumina Genome Analyzer Hiseq2500.

Sequences were aligned to the Ensemble mouse genome GRCm38 using Bowtie2 (Langmead and Salzberg, 2012) and Tophat2 (Kim et al., 2013), and counted with HTSeq-count (Anders et al., 2015). Cell debris and doublets were removed from the data by inspecting miscroscope images of the microfluidic chips. Low-quality libraries were excluded whenever their transcript abundance was less than 10^6 reads and the number of expressed genes was less than one thousand. The library sizes were then normalized to read counts per million (CPM). Genes with counts in less than 10 cells or annotated as pseudogenes were excluded from the analysis.

Dimension Reduction

The variation of gene expression contained in single cell transcriptomes is a combination of meaningful biological differences and technical noise from sampling effects due to low-numbered transcripts. We set up a dimensionality reduction strategy to identify the genes demonstrating significant variation and concerted patterns of expression.

We performed a selection of genes with concerted patterns of expression by calculating the mutual information for all pairs of gene in the dataset. Mutual information is used here as a generalized correlation measure enabling the quantification of nonlinear gene relationships. Gene levels were preliminary discretized using a nonparametric and unsupervised density estimation algorithm, Bayesian Blocks (Python module ‘‘AstroML’’). Mutual information for each pair of discretized gene levels was determined by subtracting the joint entropy from the sum of both genes’ marginal entropy (R package ‘‘entropy’’). Genes were retained if they shared sufficient mutual information with at least 2 other genes (MI > 0.25).

In order to refine gene lists and identify the dominant biological characteristics, we calculated the Spearman rank correlation matrix of gene expression for the dataset. We then grouped correlated genes into an initial set of gene modules by performing a hierarchical clustering of the correlation matrix. Because the correlated genes were more abundant in our in vitro dataset (445 genes) compared to our in vivo dataset (136 genes), we adopted a different strategy for defining the gene modules when processing the two datasets. For the in vitro dataset, the dendrogram associated with the hierarchical clustering was thresholded to obtain about 300 gene modules mostly composed of 1 or 2 genes and the larger modules (at least 4 genes) were retained. This method is similar to the one described in (Patel et al., 2014). Some modules were discarded if they did not have a recognizable function. For the in vivo dataset, the associated dendrogram was thresholded such as obtaining the 5 gene modules described in the main text.

Our pipeline does not explicitly select genes based on their variability. In order to assess whether the genes contained in the selected modules showed significant variation, we evaluated the dispersion of each gene, i.e. the ratio of variance over mean, for each gene across the single cell dataset, as described in (Satija et al., 2015). In this algorithm each gene is associated with one of 20 bins, based on their associated average expression and the dispersion measures are z-normalized within each bins. We obtained an average z-score of about 1 for our selection of genes, confirming that they indeed showed substantial variation.

Cell Population Clustering

Cell populations were identified by hierarchical clustering using R’s Ward.D2 clustering and the Spearman correlation of the z-scored gene levels of the genes comprising the selected modules (*in vivo* modules in Figure 1B, in vitro modules in Figure 3D). Hierarchical clustering was performed on the normalized in vivo gene module averages in Figure 2A: first, gene levels belonging to the same module were averaged, then z-scored by module, and eventually z-scored by cell.

Single-Cell State Graph

To investigate the dynamical changes of the transcriptional profile as cells differentiate, we developed a method to relate each cell to its most similar siblings. Unlike cluster analysis which aims to partition cells into groups with similar characteristics, hence breaking the continuity of cell state differentiation, graphs can connect individual cells without requiring the definition of groups. These structures can reveal the differentiation trajectories and intermediate states that underlie the clusters of similar cells (the ‘‘clustered’’ populations).

Using the logarithm of normalized levels (log_{10}-transcripts per million) in the reduced space, we first calculated the Euclidean distance matrix between each cell and hence constructed a complete weighted graph of cell similarity D. In (Trapnell et al., 2014, Camp et al., 2015), a minimum spanning tree algorithm (MST) was used to extract the subset of cell-cell edges which forms the backbone of differentiation branches. While MSTs ensure that all cells are connected, they are also sensitive to noise, making the local topology sensitive to small changes in the data (Zemel and Carreira-Perpinan, 2005). To improve robustness to noise of MSTs, we constructed a consensus graph which combines multiple perturbed minimum spanning trees (pMST). Each pMST is obtained by calculating a MST from the cell dissimilarity matrix D with a certain ratio j of its elements are set to a very large value (j=20%), hence forbidding the recruitment of the associated edges. Individual pMST are merged by summing their adjacency matrix A into a matrix storing the occurrences of each edge. We then exclude rare used edges by clustering the non-null edge occurrence distribution using the Fisher method (Fisher, 1958) and removing all edges belonging to the first class. This leaves edges that are used repeatedly in multiple permutations and therefore represent choices for inclusion in MST graphs. The perturb-and-merge algorithm works iteratively until convergence in the number of included edges.

Pseudo-Temporal Ordering

One of the advantages of generating a single-cell state graph is the possibility to infer a pseudotemporal ordering of the gene expression by following the gene expression implied by the spanning tree. The strategy we applied was to isolate two terminal cell populations, *early* and a *late* and identify the K-shortest paths that connect each pair of early and late cells (Martins and Pascoal, 2003). Typically, thousands of paths were generated from terminal populations consisting in 12 cells and $K = 100$. The resulting paths were not necessarily the same length hence, in order to average gene expression along all paths, we first rescaled them into a common pseudotime scale. Considering that all cells belonging to the early (late) population would define the initial (final) pseudotime point, and setting the number of pseudotime points to a value (typically 50), we simply rebinned the gene expression levels without interpolation according to each cell rank along the path. The averaged gene expression along the pseudotime line was then smoothed using a local polynomial regression fit (R function *loess* with *span*=0.5).

In order to compare the pseudotemporal ordering obtained from different cell state graphs, we defined the fractional identity of each cell as the score obtained by linear combination of the normalized gene levels of a given list of genes assumed to be either increasing or decreasing between the terminal populations. These fractional identity scores were then used to define the start and end coordinates of the timelines in a reference pseudotime axis, i.e. the developmental timeline. When comparing the e8.5 to the e9.5 cells (Figure 1C), the gene list was composed of Sox2, Nkx1.2, Tbx6, Msgn1 and Meox1 with the first two genes having a negative coefficient (equal to -1) and the last three a positive one (equal to 1) when linearly combined. When comparing the e8.5 to the D3 cells (Figure 2E), the gene list was composed of the 99 NMP signature genes with the 68 NMP markers having a coefficient equal to -1 and and the 31 mesoderm markers' coefficients equal to 1. In Figure 2E, the fractional identities were normalized independently for in vivo and in vitro cells, leading to equal-length timelines.

Deterministic Model of Gene Regulation

A gene regulation model based on a thermodynamic formulation (Sherman and Cohen, 2012) was used to simulate the gene expression dynamics of the Bra-Sox2-Msgn1/Tbx6 network.

This ordinary differential equation (ODE) model describes that the production rate of a gene X as function of the occupation state of its enhancer and is expressed as the product of p_X^{bound}, the probability of RNA polymerase to be bound to the promoter and α_X and the maximal production rate of the gene.

To calculate the polymerase binding probability, all possible states of the enhancer are enumerated, i.e. all possible combinations of upstream transcription factors (TFs) bound or not bound to the gene's cis-regulatory modules (CRM). Let S_X be the set of inputs binding to the CRM of X.

$$S_X = \{Pol\} \cup \{Act\} \cup \{Rep\} \quad \text{(Equation 1)}$$

where $\{Pol\}$ represents the RNA polymerase, $\{Act\}$ is the set of activating transcription factors and $\{Rep\}$ is the set of repressing TFs upstream of X. In order to account for the situation of transcription factors being bound independently to multiple sites, multiple instances of the same TF can be stored in $\{Act\}$ and $\{Rep\}$.

Then, each possible promoter state s is represented by an element of the powerset of S_X, and is associated with a statistical weight $W(s)$ derived from Gibbs free energy of each binding interaction (Shea and Ackers, 1985).

$$\forall s \in \mathscr{P}(S_X), W(s) = \prod_{T \in s} K_{TX} c_{TX} x_T \quad \text{(Equation 2)}$$

where K_{TX} is the equilibrium binding constant of input T on X, c_{TX} is the level of binding cooperativity between the input T and RNA polymerase, and x_T is the concentration of T. When $c_{TX}>1$, T acts as an activator and conversely, if $c_{TX}<1$, T acts as a repressor. In the following, we assume that all repressors are strongly inhibiting polymerase binding, i.e. $c_{TX} = 0, \forall T \in \{Rep\}$. We denote by $(S_X)^+$ the set of transcriptionally active states, i.e. containing RNA polymerase and no repressor, and $(S_X)^-$ the other transcriptionally inactive states.

The polymerase binding probability is defined as the ratio of Z_X^{bound}, the sum of the statistical weights of all transcriptionally active states over the total statistical weight of all possible states.

$$p_X^{\text{bound}} = \frac{Z_X^{\text{bound}}}{Z_X^{\text{unbound}} + Z_X^{\text{bound}}} \quad \text{(Equation 3)}$$

The factored forms of the total statistical weight of all active and inactive states read:

$$Z_X^{\text{bound}} = \sum_{s \in \mathscr{P}(S_X)^+} W(s) = K_{PolX} x_{Pol} \prod_{T \in \{Act\}} (1 + K_{TX} c_{TX} x_T) \quad \text{(Equation 4)}$$

$$Z_X^{\text{unbound}} = \sum_{s \in \mathscr{P}(S_X)^-} W(s) = \prod_{T \in \{Act\} \cup \{Rep\}} (1 + K_{TX} x_T)$$

As an example, the complete statistical weights of the genes interacting according to the best topology shown in Figure 7C read:

$$\text{Sox2}\begin{cases} Z_S^{\text{bound}} = K_{PolS}x_{Pol}(1+K_{RS}c_{RS}x_R)^{n_{RS}} \\ Z_S^{\text{unbound}} = (1+K_{RS}c_{RS}x_R)^{n_{RS}}(1+K_{BS}c_{BS}x_B)^{n_{BS}}(1+K_{TS}c_{TS}x_T)^{n_{TS}} \end{cases} \quad \text{(Equation 5)}$$

$$\text{Bra}\begin{cases} Z_B^{\text{bound}} = K_{PolB}x_{Pol}(1+K_{WB}c_{WB}x_W)^{n_{WB}} \\ Z_B^{\text{unbound}} = (1+K_{WB}c_{WB}x_W)^{n_{WB}}(1+K_{SB}c_{SB}x_S)^{n_{SB}}(1+K_{TB}c_{TB}x_T)^{n_{TB}} \end{cases}$$

$$\text{Msgn1/Tbx6}\begin{cases} Z_M^{\text{bound}} = K_{PolM}x_{Pol}(1+K_{BM}c_{BM}x_B)^{n_{BM}} \\ Z_M^{\text{unbound}} = (1+K_{BM}c_{BM}x_B)^{n_{BM}}(1+K_{SM}c_{SM}x_S)^{n_{SM}} \end{cases}$$

where *S* stands for Sox2, *B* for Bra, *M* for Msgn1/Tbx6, *R* for RA, *W* for Wnt and *Pol* for RNA polymerase; and n_{TX} represent the number of binding sites for TF *T* onto gene *X*'s CRM.

The rate of change of protein concentration is given by the first order rate equation:

$$\dot{x}_X = \alpha_X p_X^{\text{bound}} - \delta_X x_X \quad \text{(Equation 6)}$$

where δ_X is the degradation rate of protein X.

Finally, to account for the external conditions to which the cells are exposed, we introduce two extrinsic concentration terms, x_R^{ext} for RA and x_W^{ext} for Wnt, which are added to the previous concentration terms when these pathways are activated.

Optimization Algorithm

Our optimization strategy aims at evaluating the fitness of all network topologies linking the 3 TF nodes Bra, Sox2 and Msgn1/Tbx6. Allowing each of the 6 interactions to act as an activator or as a repressor, the parameter space of $2^6 = 64$ topologies was explored. We used a Python implementation of the Differential Evolution algorithm (DE) (Storn and Price, 1997) (Python function *optimize.differential_evolution* from the *SciPy* module). DE is a simple and fast evolutionary algorithm well suited for high-dimensional optimization, but it does not guarantee an optimal solution. To strengthen our confidence in the solutions identified, each topology optimization was run independently 30 times from random initial populations, hence generating a set of 30 best individuals. This population of best-fit solutions was then clustered into one or more subgroups of similar parameter signatures. The clustering was processed by fitting Gaussian Mixture Models with an expectation-maximization algorithm and identifying the optimal number of clusters according to the Bayesian information criterion for each model. Each topology run offered at least one optimal cluster of at least 4 individuals from which the average topology performance was calculated.

Parameters, Initial State and External Conditions

The model parameters were specified according to the following rules:

- Each topology is defined by setting the cooperativity factor c_{TX} to 10 for activators, and 0 for repressors.
- For each topology, the only optimized parameters are the 6 binding affinities K_{TX}, i.e. K_{MB}, K_{SB}, K_{BS}, K_{MS}, K_{BM}, K_{SM}. These parameters are constrained in the [0.01,1000] interval.
- Wnt and RA binding affinities are kept constant, $K_{WB} = K_{RS} = 10$
- RNA polymerase binding affinity is kept constant, $K_{PolX} = 1$.
- The number of binding sites is set to 2 ($n_{TX} = 2$).
- The maximal production and degradation rates of each protein is set to 2 ($\alpha_X = \delta_X = 2$) which constrains protein concentrations to the [0,1] interval.

At the initial D2 time point, all initial protein concentrations are close to 0: $x_B(t_{D2}) = x_M(t_{D2}) = x_R(t_{D2}) = x_W(D2) = 0.001$ except for Sox2 concentration which is high, $x_S(t_{D2}) = 0.8$. This represent the "epiblast" state.

During the simulations, the cells are exposed to one of the four signaling conditions (see dashed lines in Figure 7A):

Mesodermal condition: Wnt high ($x_W^{\text{ext}} = 1.0$) and basal level of RA ($x_R^{\text{ext}} = 0.1$) from D2 to D5.
Neural condition I (RAplus): same external concentrations between D2 and D3 as above, then RA high ($x_R^{\text{ext}} = 1.0$) and null Wnt ($x_W^{\text{ext}} = 0.0$) between D3 and D5.
Neural condition II (NB): same external concentrations between D2 and D3 as above, then basal RA ($x_R^{\text{ext}} = 0.1$) and null Wnt ($x_W^{\text{ext}} = 0.0$) between D3 and D5.
Anterior condition: basal level of Wnt and RA ($x_W^{\text{ext}} = x_R^{\text{ext}} = 0.1$) between D2 and D5.

Finally, the Brachyury mutant and Msgn1/Tbx6 mutant simulations were performed with the same parameters as the WT simulations, the only difference being the elimination of the associated protein by setting its maximum production rate to 0 ($\alpha_B = 0$ or $\alpha_M = 0$).

Distance Function

We defined a distance function to quantitatively evaluate the differences between the simulated dynamics and reconstructed WT and mutant timepoints. Each parametric solution was simulated six times: 3 WT *experiments* (Mesodermal, Neural I and Anterior conditions) and 1 Bra mutant *experiment* (Mesodermal condition) and 2 Msgn1/Tbx6 mutant *experiments*. After scaling the trajectories of each gene by dividing by the maximum value for that gene over all 6 experiments, each experiment score was calculated by averaging the absolute difference between the simulated and targeted gene levels at specific time points (squares in Figure 7A). The distance function is then obtained by summing these 6 experiment scores.

Stochastic Model of Gene Regulation

To investigate how the the minimal network model behaves when challenged with the inherent stochasticity of gene regulation, we converted each gene rate equation (Equation 6) to follow the Chemical Langevin Equation (CLE) approximation:

$$\dot{x}_X = \alpha_X p_X^{\text{bound}} - \delta_X x_X + \sqrt{\frac{\alpha_X p_X^{\text{bound}} + \delta_X x_X}{\Omega}}\xi \qquad \text{(Equation 7)}$$

where Ω is the volume parameter relating concentrations with number of molecules ($N_X = x_X\Omega$) , and ξ is a random variable following a normal distribution with zero mean and unit variance.

The transformation procedure is detailed in (Perez-Carrasco et al., 2016).

QUANTIFICATION AND STATISTICAL ANALYSIS

To quantify immunofluorescence intensities in fixed cells all images were converted to 8-bit images and were segmented in classes by thresholding using methods available in FIJI. The signal area of each marker was calculated and divided by the corresponding total signal area for DAPI.

All bar plots' error bars indicate the standard deviation of randomly selected independent fields for immunofluorescence quantifications or the standard deviation of biological replicates for qRT-PCR quantifications. Sample size are indicated in the associated figure legends.

Genes differentially expressed between distinct cell populations were assessed by performing an approximate χ^2 likelihood ratio test between a Tobit model fitted to the two populations (alternative model) and a Tobit model fitted to all cells (null model), using the *differentialGeneTest* function of the Monocle package (Trapnell et al., 2014). This test was performed independently for each gene and selected sets were obtained by thresholding on p-values (p-values threshold of 5e-5 for Figures 1E, 1F, 1G and 5e-7 for Figures 6I, 6J).

All other details of the single cell RNA-seq analysis are provided in the detailed methods.

DATA AND SOFTWARE AVAILABILITY

The accession number for the single cell RNA-seq data reported in this paper is ArrayExpress: [E-MTAB-5208].

All simulations and analysis were performed in R (The R Foundation) and Python (Python Software Foundation) as described in the detailed methods.

Developmental Cell, Volume *41*

Supplemental Information

A Gene Regulatory Network

Balances Neural and Mesoderm Specification

during Vertebrate Trunk Development

Mina Gouti, Julien Delile, Despina Stamataki, Filip J. Wymeersch, Yali Huang, Jens Kleinjung, Valerie Wilson, and James Briscoe

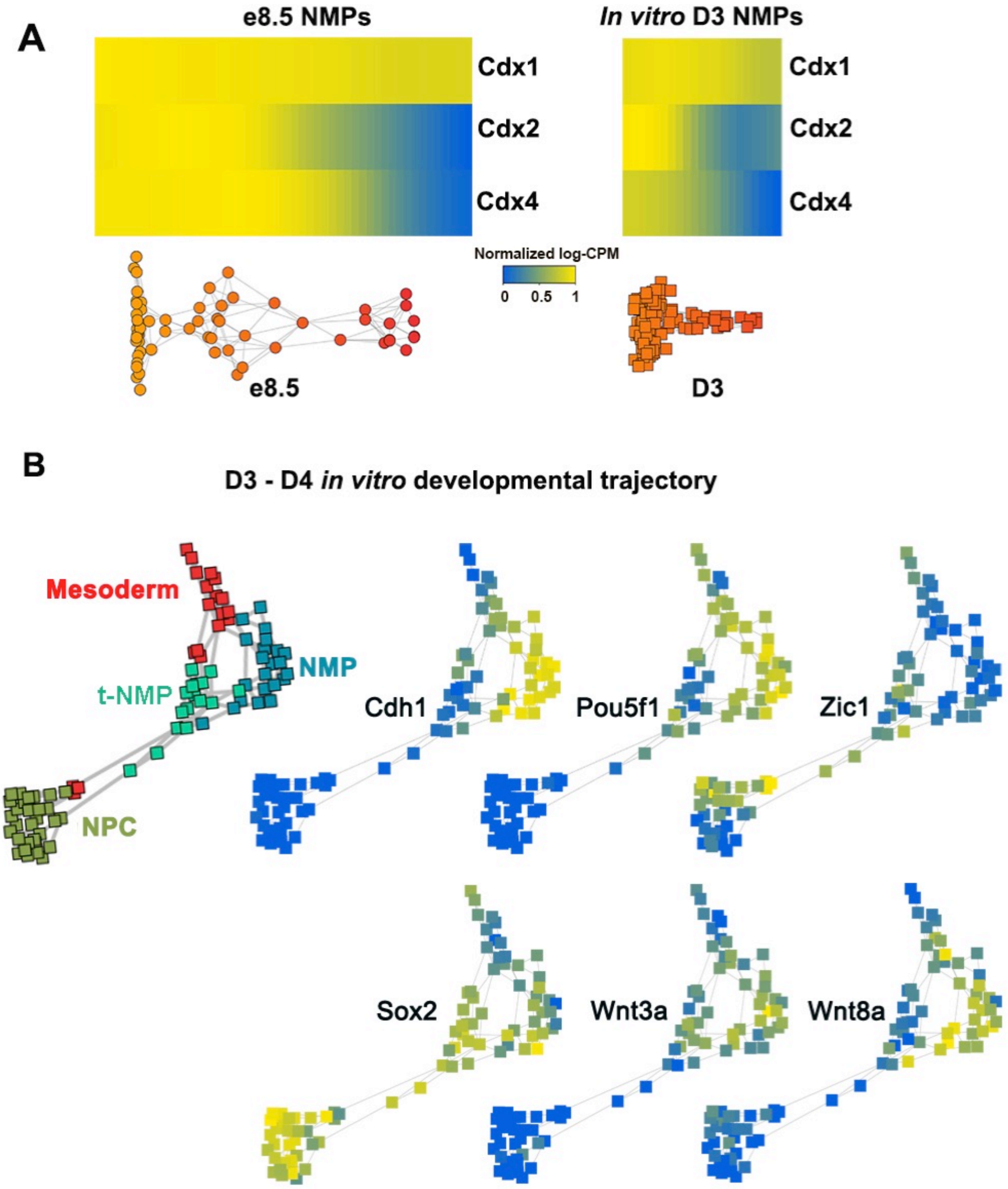

Figure S1. Temporal expression of differentially regulated genes during NMP differentiation. Related to Figure 3. (A) Comparison of *Cdx* expression (*Cdx1*, *Cdx2* & *Cdx4*) in e8.5-NMPs and *in vitro* D3 NMPs shows a similar expression pattern. *Cdx1* expression is maintained in cells differentiating to PSM both *in vivo* and *in vitro* whereas the expression of *Cdx2* and *Cdx4* is downregulated. Expression values for each gene are shown as the log of normalized counts per million reads (log-CPM). (B) *Cdh1* is specifically expressed in early NMPs (similar to e8.5-NMPs) whereas *Wnt3a* is expressed in early NMPs and t-NMPs. *Pou5f1* (*Oct3/4*) and *Wnt8a* is expressed in NMPs and is downregulated as cells progress to the t-NMP or NPC. *Zic1* and *Sox2* is upregulated as cells differentiate to NPCs.

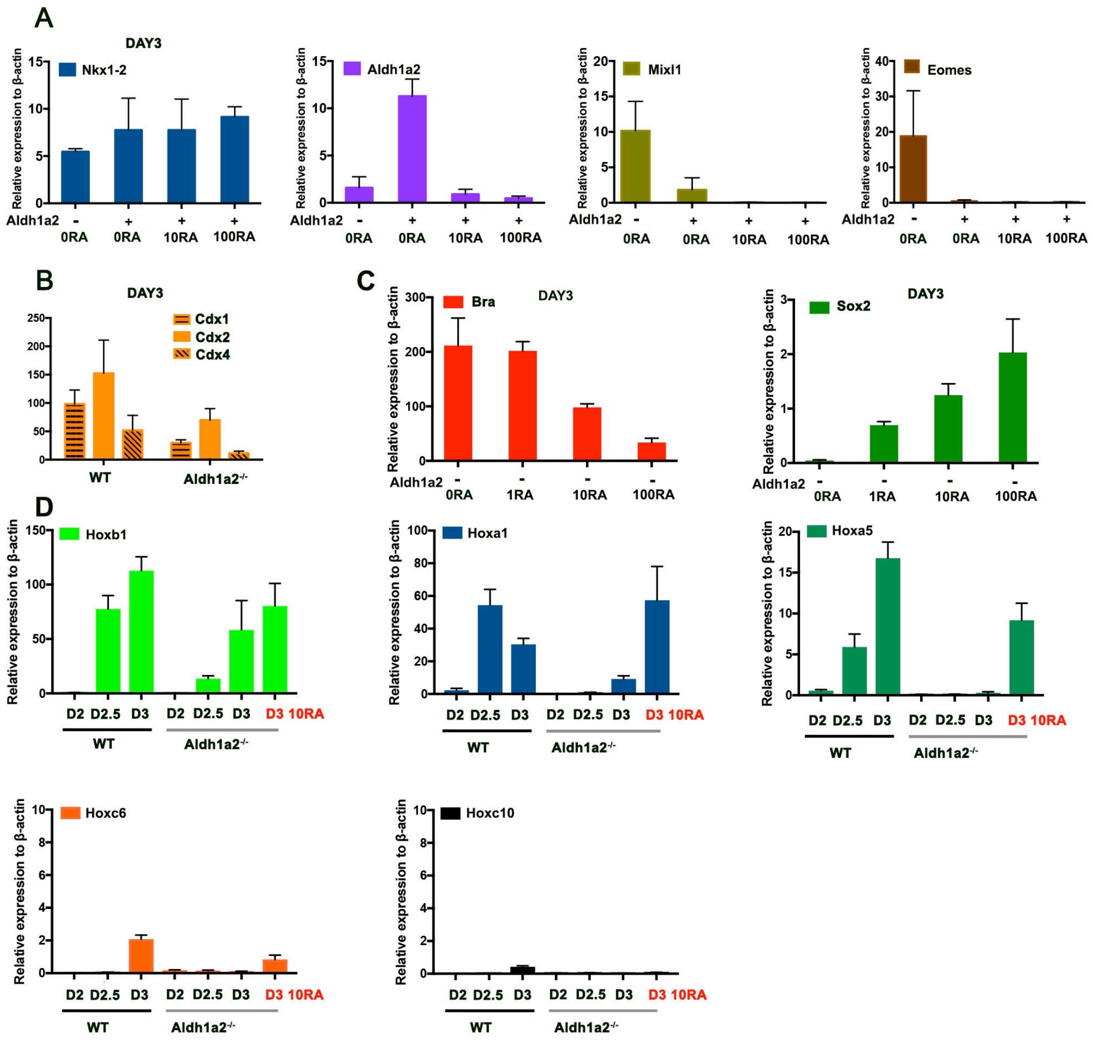

Figure S2. Induction of NMPs requires basal levels of RA signalling. Related to Figure 4. (A) qRT-PCR analysis of the expression of *Nkx1.2*, *Aldh1a2*, *Mixl1* and *Eomes* at D3 in *Aldh1a2*$^{-/-}$ and WT cells treated with CHIR/FGF or WT cells treated with CHIR/FGF/RA (RA 10nM or 100nM). Expression of *Nkx1.2* is similar in all conditions, whereas expression of *Aldh1a2* is high in WT cells at D3 but *Mixl1* and *Eomes* are induced in *Aldh1a2*$^{-/-}$ cells under CHIR/FGF conditions. (B) The expression of *Cdx1*, *Cdx2* and *Cdx4* is downregulated in the *Aldh1a2*$^{-/-}$ cells at D3. (C) qRT-PCR analysis of the expression of T/*Bra* and *Sox2* in the *Aldh1a2*$^{-/-}$ cells treated with CHIR/bFGF and different concentrations of RA (1nM, 10nM or 100nM). (D) Analysis of Hox gene expression at D2, D2.5 and D3 of differentiation revealed that anterior *Hox* genes like *Hoxb1* are induced in the absence of RA signalling (*Aldh1a2*$^{-/-}$ cells), whereas the expression of more posterior *Hox* genes like *Hoxa5* and *Hoxc6* is RA dependent. Addition of 10nM RA between D2-D3 in the *Aldh1a2*$^{-/-}$ cells rescues the expression of *Hoxa5* and *Hoxc6*.

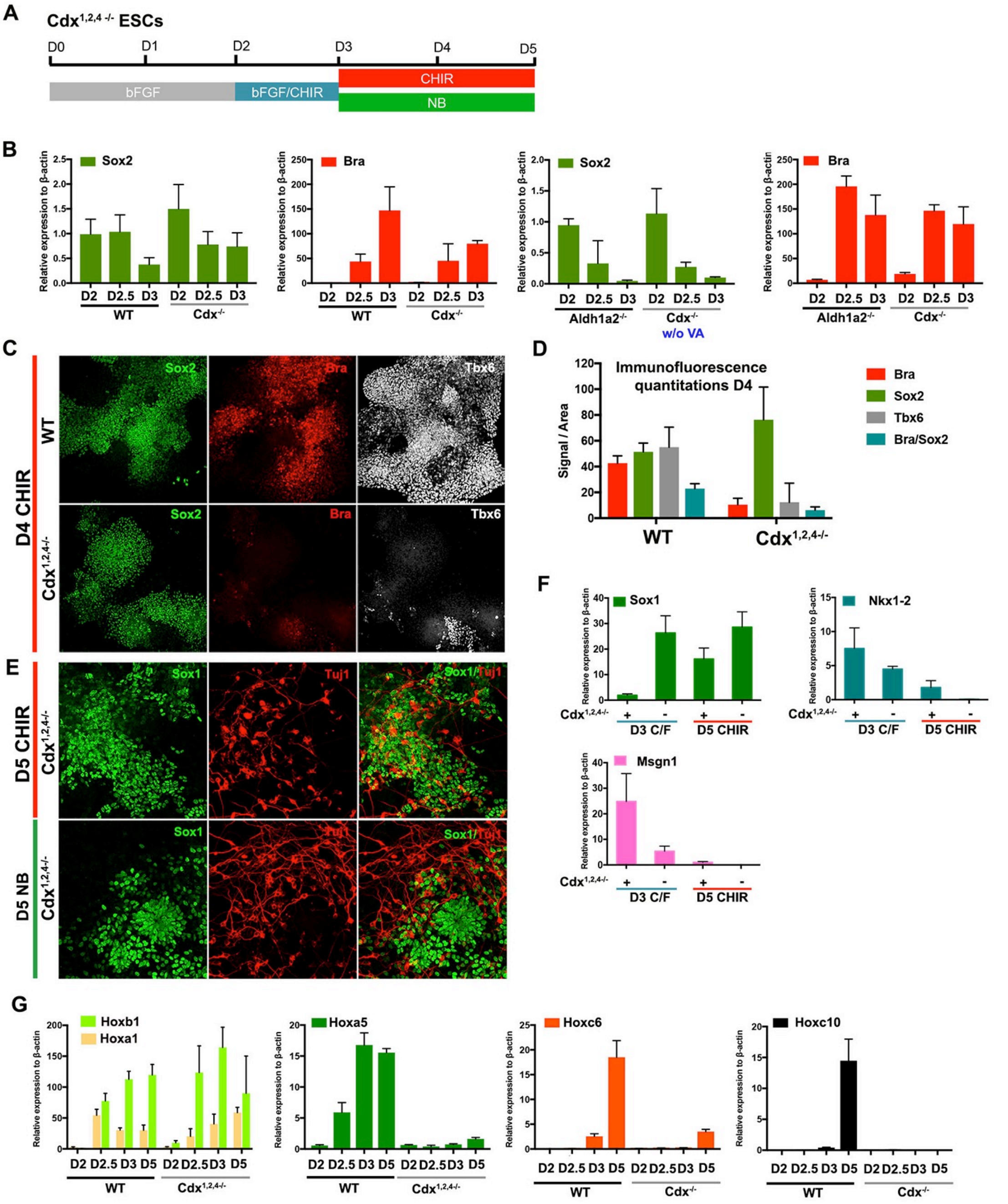

Figure S3. **Cdx genes are required for the establishment of NMP identity and induction of trunk Hox genes. Related to Figure 4.** (A) Schematic of *in vitro* differentiation conditions of *Cdx*$^{1,2,4-/-}$ cells. (B) qRT-PCR analysis of *Sox2* and *T/Bra* levels at D2, D2.5 and D3 of differentiation in WT and *Cdx*$^{1,2,4-/-}$ cells shows similar response during the early stages of differentiation (D2, D2.5) whereas at D3 the expression of *T/Bra* is significantly lower in the *Cdx*$^{1,2,4-/-}$ cells. In the absence of RA signalling (Vitamin A depleted conditions) the expression of *Sox2* is significantly reduced and *T/Bra* expression is induced at an earlier time point (D2.5) in both *Aldh1a2*$^{-/-}$ and *Cdx*$^{1,2,4-/-}$ cells. (C) At D4 of differentiation (CHIR conditions), the expression of *T/Bra* is downregulated in *Cdx*$^{1,2,4-/-}$ cells, which acquire a NPC identity. (D) Quantitation of T/Bra^{+}, Sox2^{+}, Tbx6^{+} or T/Bra^{+}/Sox2^{+} signal^{+} area normalized to DAPI area at day4 (CHIR) of differentiation in WT and *Cdx*$^{1,2,4-/-}$ cells. Error bars indicate s.d, of randomly selected independent fields n≥4. (E) At D5 *Cdx*$^{1,2,4-/-}$ cells predominantly differentiate to neural cells expressing Sox1 and Tuj1 in both CHIR and NB conditions. (F) qRT-PCR analysis of *Sox1*, *Nkx1.2* and *Msgn1* at D3 (NMP conditions) and D5 (CHIR conditions) verifies the inductions of neural identity in the *Cdx*$^{1,2,4-/-}$ cells. (G) Analysis of *Hox* gene expression at D3 and D5 (CHIR conditions) of differentiation revealed that *Cdx*$^{1,2,4-/-}$ cells express *3' Hox* genes, such as *Hoxb1*, whereas WT cells acquire a progressively more posterior identity characterized by the expression of *Hoxa5*, *Hoxc6*, *Hoxc9* and *Hoxc10* at D5.

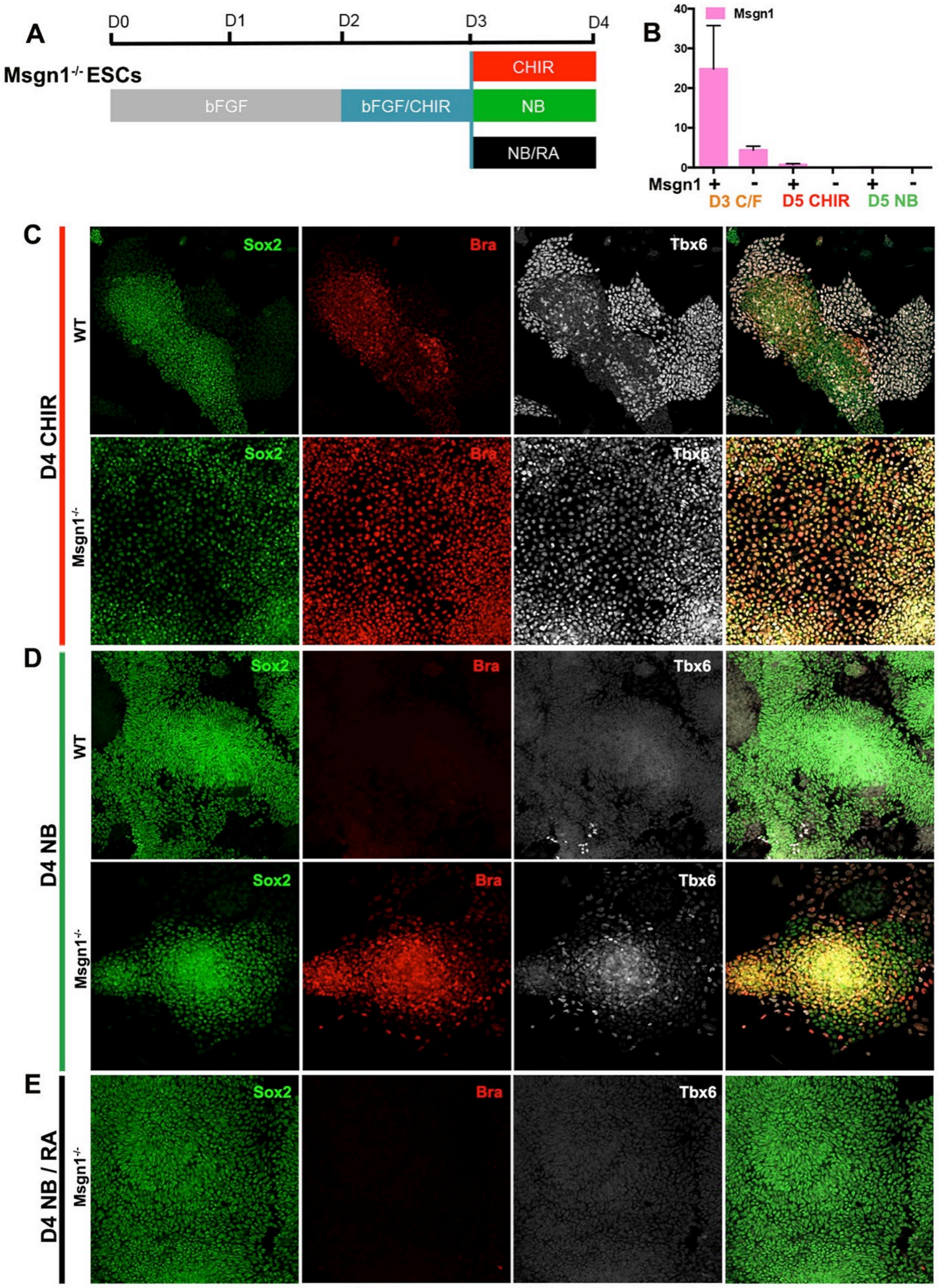

Figure S4. Msgn1 is required for the MPC to PSM transition. Related to Figure 5. (A) Schematic of *in vitro* differentiation conditions of *Msgn1*$^{-/-}$ cells. (B) Expression of *Msgn1* is induced at D3 in the WT cells exposed to CHIR/FGF. (C) At D4 in CHIR conditions, *Msgn1*$^{-/-}$ cells co-express T/Bra/Sox2 with Tbx6 whereas WT cells express Tbx6 but not T/Bra. (D) After removal of CHIR/FGF, in NB conditions, the expression of T/Bra is maintained longer in the *Msgn1*$^{-/-}$ cells compared to WT. Treatment with 100nM RA from D3 to D4 resulted in the downregulation of T/Bra in *Msgn1*$^{-/-}$ cells.

Table S5. Oligonucleotides used in the study, related to STAR Methods.

Gene	Primer Sequence (5'-3')
Actin F	TGGCTCCTAGCACCATGA
Actin R	CCACCGATCCACACAGAG
Eomes F	GGCCTACCAAAACACGGATATC
Eomes R	TTTCTGAAGCCGTGTACATGGA
Mixl1 F	CTACCCGAGTCCAGGATCCA
Mixl1 R	ACTCCCCGCCTTGAGGATAA
Nkx1.2 F	ACTGCCTTCACTTACGAGCA
Nkx1.2 R	AAATTTTGACCTGCGTCTCC
T/Bra F	ACACACGGCTGTGAGAGGTA
T/Bra R	TTATCATGGGACTGCAGCAT
Cdx1 F	ACAGCCGGTACATCACTATCC
Cdx1 R	CTTGTTTACTTTGCGCTCCTTG
Tbx6 F	CCCAACTATGCAGCCAACACT
Tbx6 R	CTGTGTGATCCTAGGGTTCTGGTA
Msgn1 F	CTTCTGACACCGCTGGTCTG
Msgn1 R	GTGACTGCCGTAGCCATCG
Hoxb1 F	AGAGGCTGGCTTACGAGAC
Hoxb1 R	GGTTGAGGCTTGCTTGAGG
Hoxc6 F	CAGGTAAAGGCAAAGGGATG
Hoxc6 R	ATAGGCGGTGGAATTGAGG
Hoxc9 F	AGCCGACAGAGACAGATTAC
Hoxc9 R	AATGCCAGTCCCAGAAGC
Hoxc10 F	GTCCAGACACCTCGGATAAC
Hoxc10 R	AATGGTCTTGCTAATCTCCAG
Hoxa13 F	AAATGTACTGCCCCAAAGAG
Hoxa13 R	GATATCCTCCTCCGTTTGTC
Hoxc13 F	GTCAGGTGTACTGCTCCAAG
Hoxc13 R	CCTTCTCTAGCTCCTTCAGC
Cdx2 F	TAGTCGATACATCACCATCAGG
Cdx2 R	TGATTTTCCTCTCCTTGGCTCT
Cdx4 F	GCAATAGATACATCACCATCAGG
Cdx4 R	ACTTTGCACGGAACCTCCAG
Fgf8 F	GCTCATTGTGGAGACCGATA
Fgf8 R	AATACGCAGTCCTTGCCTTT
Wnt3a F	ATGGCTCCTCTCGGATACCT
Wnt3a R	GGGCATGATCTCCACGTAGT
Aldh1a2 F	ATGGGTGAGTTTGGCTTACG
Aldh1a2 R	GGTTCATTGGAAGGCAGAAA
Sox1 F	AGCGTGCCTTTGATTTCTCT
Sox1 R	GGGATAAGACCTGGGTGAGA
Cyp26a1 F	GCAGGAAATACGGCTTCATC
Cyp26a1 R	ATCACCTTCTTTCGCTGCTT
Sox2 F	GGCAGCTACAGCATGATGCAGGAGC
Sox2 R	CTGGTCATGGAGTTGTACTGCAGG
